# The value of FDG PET/CT imaging in outcome prediction and response assessment of lymphoma patients treated with immunotherapy: a meta-analysis and systematic review

**DOI:** 10.1007/s00259-022-05918-2

**Published:** 2022-08-06

**Authors:** Zahra Kiamanesh, Narjess Ayati, Ramin Sadeghi, Eliza Hawkes, Sze Ting Lee, Andrew M. Scott

**Affiliations:** 1 Nuclear Medicine Research Center, Mashhad University of Medical Sciences, Mashhad, Iran; 2 Department of Nuclear Medicine, Ultrasound & PET, Sydney Westmead Hospital, Sydney, NSW Australia; 3 Olivia Newton-John Cancer Research Institute and School of Cancer Medicine, La Trobe University, Victoria, Australia; 4 Department of Medicine, University of Melbourne, Victoria, Australia; 5 Department of Medical Oncology & Clinical Haematology, Austin Health, Heidelberg, VIC Australia; 6 School of Public Health & Preventative Medicine, Monash University, Melbourne, Australia; 7 Department of Molecular Imaging & Therapy, Austin Health, 145 Studley Road, Heidelberg, VIC 3084 Australia

**Keywords:** ^18^F-FDG PET/CT, Immunotherapy, Malignant lymphoma, Anti-CD20 antibodies, Immune checkpoint inhibitors

## Abstract

**Purpose:**

Treatment strategies of lymphoid malignancies have been revolutionized by immunotherapy. Because of the inherent property of Hodgkin lymphoma and some subtypes of non-Hodgkin lymphoma as a highly FDG-avid tumor, functional ^18^F-FDG PET/CT imaging is already embedded in their routine care. Nevertheless, the question is whether it is still valuable in the context of these tumors being treated with immunotherapy. Herein, we will review the value of ^18^F-FDG PET/CT imaging lymphoid tumors treated with immunotherapy regimens.

**Methods:**

A comprehensive literature search of the PubMed database was conducted on the value of the ^18^F-FDG PET/CT for immunotherapy response monitoring of patients with malignant lymphoma. The articles were considered eligible if they met all of the following inclusion criteria: (a) clinical studies on patients with different types of malignant lymphoma, (b) treatment with anti-CD20 antibodies, immune checkpoint inhibitors or immune cell therapies, (c) and incorporated PET/CT with ^18^F-FDG as the PET tracer.

**Results:**

From the initial 1488 papers identified, 91 were ultimately included in our study. In anti-CD20 therapy, the highest pooled hazard ratios (HRs) of baseline, early, and late response monitoring parameters for progression-free survival (PFS) belong to metabolic tumor volume (MTV) (3.19 (95%CI: 2.36–4.30)), maximum standardized uptake value (SUVmax) (3.25 (95%CI: 2.08–5.08)), and Deauville score (DS) (3.73 (95%CI: 2.50–5.56)), respectively. These measurements for overall survival (OS) were MTV (4.39 (95%CI: 2.71–7.08)), DS (3.23 (95%CI: 1.87–5.58)), and DS (3.64 (95%CI: 1.40–9.43)), respectively. Early and late ^18^F-FDG PET/CT response assessment in immune checkpoint inhibitors (ICI) and immune cell therapy might be an effective tool for prediction of clinical outcome.

**Conclusion:**

For anti-CD20 therapy of lymphoma, the MTV as a baseline ^18^F-FDG PET/CT-derived parameter has the highest HRs for PFS and OS. The DS as visual criteria in early and late response assessment has higher HRs for PFS and OS compared to the international harmonization project (IHP) visual criteria in anti-CD20 therapy. Early changes in ^18^F-FDG PET parameters may be predictive of response to ICIs and cell therapy in lymphoma patients.

**Supplementary Information:**

The online version contains supplementary material available at 10.1007/s00259-022-05918-2.

## Introduction

Treatment strategies of the lymphoid malignancies were revolutionized by immunotherapy in two major advances over the last 3 decades. In 1997, the introduction of anti-CD20 monoclonal antibodies (mAbs) such as rituximab which targeted B-cells exclusively to evoke a direct anti-tumor cytotoxic effect revolutionalized the treatment paradigm for lymphoma. Then, in 2017, the introduction of immune checkpoint inhibitors (ICI) such as anti-PD1 and anti-CTLA4 monoclonal antibodies which stimulates the immune system via T cells appears to be the next promising step in lymphoma management [1–3]. Although immunotherapy now is common terminology for the class of drugs that stimulate the immune system to indirectly target cancer cells, conventionally, immunotherapy refers to any therapy that manipulates the immune system such as both anti-CD20 mAbs and new targeted immunotherapy, ICIs, and cell therapy.

Anti-CD20 monoclonal antibodies have FDA approval for treatment of non-Hodgkin lymphoma (NHL) for multiple indications as monotherapy or in combination with other lymphoma-directed therapeutics [4, 5], and have a place in treatment paradigms for many B cell lymphomas [6]. Among the immune checkpoint inhibitors, pembrolizumab and nivolumab have regulatory approval for the treatment management of the relapsed or refractory transplant ineligible or post-transplant relapsed classical Hodgkin lymphoma (HL) and relapsed or refractory primary mediastinal B cell lymphoma (PMBL) [7, 8]. CD19-directed chimeric antigen receptor (CAR) T cell therapy is approved for the treatment of certain types of large B-cell lymphoma relapsed or refractory to at least two other treatment regimens [9].

Because of the inherent property of HL and most subtypes of NHL as highly ^18^F-FDG avid tumors, functional ^18^F-FDG PET/CT imaging is a standard response assessment tool for these diseases and has a decisive role in noninvasive response monitoring of therapies, including immunotherapeutics [10, 11].

In the abovementioned framework, multiple different visual ^18^F-FDG PET criteria were introduced to more consistently evaluate ^18^F-FDG PET scans [10, 12]. Despite the available ^18^F-FDG PET criteria, following the introduction of ICIs some modified response assessment criteria were proposed. These criteria were developed based on the observation that some immunomodulatory drugs can alter tumoral glucose metabolism, changing the assumed association between the ^18^F-FDG uptake and treatment efficacy observed under conventional chemotherapy [2].

As evident from the literature, existing original articles have some differences in the population and methodologies. Specifically, the malignant lymphoma analysed (either HL or NHL), immunotherapy regimens and line of therapy (anti-CD20 mAbs, ICI versus immune cell therapy), and time points and time intervals of performing ^18^F-FDG PET scan (baseline, early, and late ^18^F-FDG PET imaging), all vary considerably.

Various studies have dealt with the issue of the value of metabolic imaging by ^18^F-FDG PET scan for immunotherapy management in malignant lymphoma patients, but the evidence for lymphoma treated with ICIs is lacking due to the current standard being that of a quantitative or semi-quantitative assessment, primarily using the Lugano or LYRIC criteria [13, 14]. Here, we will review the role of ^18^F-FDG PET imaging in response assessment or response prediction in the lymphoid tumors treated with immunotherapy regimens using the perspective of the mentioned studies.

## Methods

### Literature search

A comprehensive literature search of the PubMed database was conducted to retrieve relevant published articles concerning the value of the ^18^F-FDG PET/CT for response monitoring of patients with malignant lymphoma to immunotherapy including anti-CD20 therapy, ICI therapy, and cell therapy. The search was based on the various combinations of the Boolean operators and the following keywords “lymphoma,” “Hodgkin disease,” “Hodgkin lymphoma,” “non-Hodgkin lymphoma,” “^18^F-FDG PET/CT,” “positron emission tomography,” “immunotherapy,” “immune checkpoint inhibitors,” “anti-CD20 therapy,” “CAR T cell therapy,” “Rituximab,” “Nivolumab,” “Ipilimumab,” “Pembrolizumab”…. No date or language restriction was applied and the search was updated until March 2021. The reference list of the eligible articles was manually screened to identify any pertinent study.

### Eligibility criteria

The relevant original articles were considered eligible if they met all of the following inclusion criteria: (a) clinical studies on patients with different types of malignant lymphoma including Hodgkin disease and non-Hodgkin lymphoma; (b) treatment with anti CD-20 mAbs, ICIs, or cell therapy; and (c) incorporation of PET/CT with the ^18^F-FDG as the PET tracer. The exclusion criteria were as follows: (a) investigations on animals, (b) radioimmunotherapy as treatment, (c) PET/CT imaging with PET tracers other than ^18^F-FDG, (d) articles without sufficient data regarding performed ^18^F-FDG PET/CT, (e) duplicated articles, (f) CNS lymphoma due to physiologic high ^18^F-FDG uptake in the central nervous system, and (g) HIV-related lymphoma.

### Data extraction and quality assessment

The required study characteristics were extracted by reviewing the whole text of the eligible articles. The gathered data were arranged in three main parts: basic study characteristics including the name of the first author and publication date; demographic information including the number of participants, lymphoma subtype, and immunotherapy regimen; and the technical aspects including ^18^F-FDG PET imaging method and findings, response assessment criteria or technique, outcomes, and hazard ratios (HR). In the cases that HR was not directly reported using depicted Kaplan–Meier curve, Graph Digitizer version 2.24, Richard Steven’s excel workbook, the HR and its 95% CI were estimated. The quality of all eligible articles was evaluated by employing the established critical appraisal tool obtained from the Oxford Center for Evidence-Based Medicine [15]. This tool was designed to evaluate the quality of the prognostic studies by taking into account several factors consisting of patient registration time, follow-up duration, outcome criteria, and adjustment for important prognostic factors. All quality assessments have been tabulated in the supplemental table 1 [16–106].

For metabolic baseline parameters, we considered tumor burden indices, including metabolic tumor volume (MTV), and total lesion glycolysis (TLG), and tumor metabolism indices, including maximum of standardized uptake value (SUVmax). For the response assessment we considered visual methods, including Deauville score (DS) and ΔSUVmax as a semi-quantitative method [21, 22, 48, 67, 77, 80, 89, 104].

### Statistical analysis

The statistical analyses for pooling hazard ratios were carried out using Comprehensive meta-analysis software (CMA version 2). The random effects model was used to pool effect sizes across included studies. Heterogeneity was evaluated using Cochrane *Q* value (*p*-values less than 0.05 were considered statistically significant) and *I*^2^ index. Publication bias was evaluated graphically using funnel plots. Because of discrepancies in methodological aspects of the included articles, the evaluation of publication bias was not possible for all papers, and only was performed for studies with similar reported indices (for more details see supplemental figures file).

## Results

**Fig. 1:**
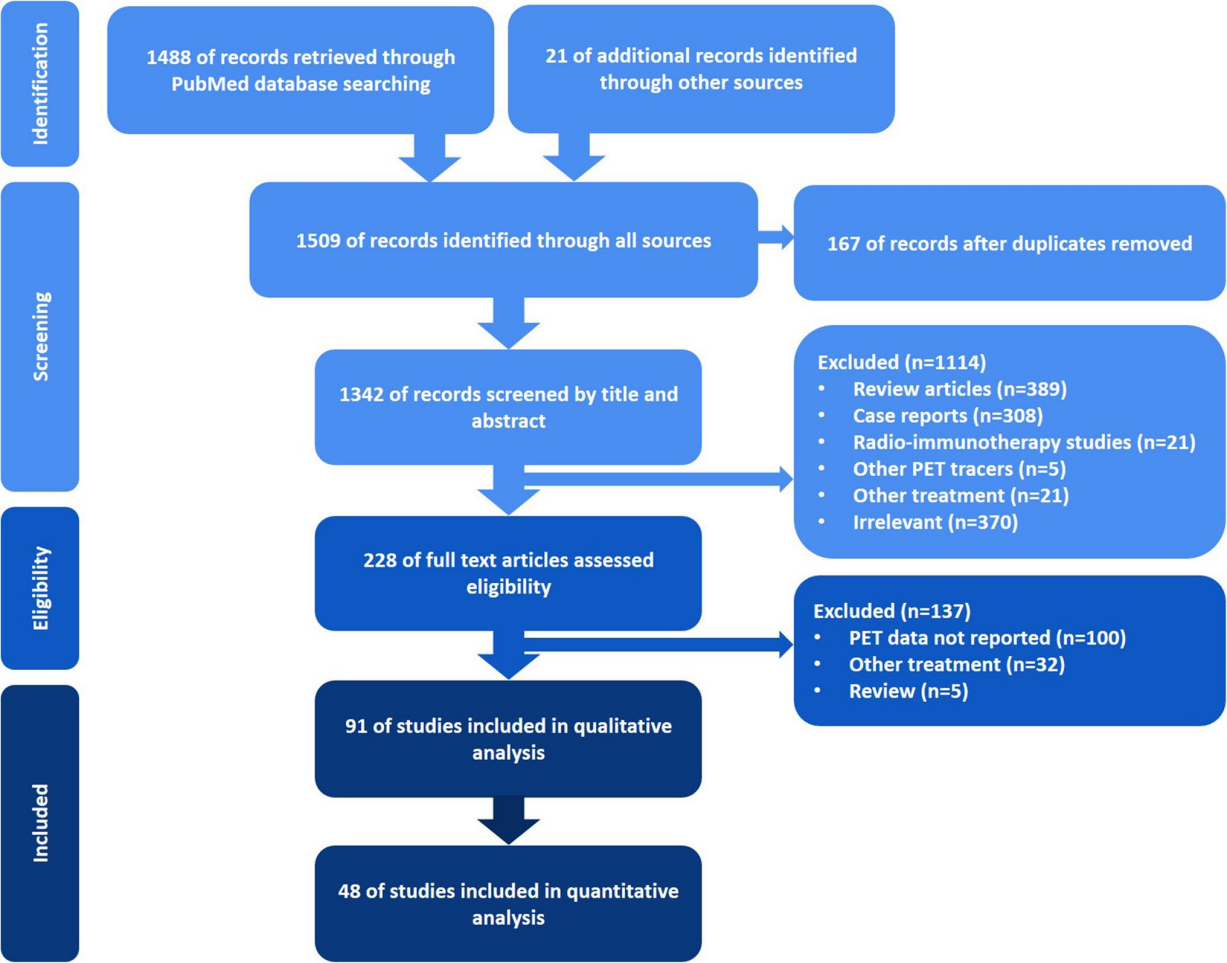
The PRISMA flowchart of performed search approach

The performed search approach is presented as a PRISMA flowchart [107] in Fig. 1. From the initial 1488 identified papers, 91 were ultimately included in our study. As we expected, the eligible included studies showed discrepancies in methodological aspects and the main remarkable one was the applied treatment strategy. In the first step, the enrolled studies were classified into studies on anti-CD20 monoclonal antibodies (for example, rituximab), studies on ICIs (for example, nivolumab), and studies on cellular therapies (for example, CAR T-cell therapy or dendritic cell therapy). The former part involved 77 publications, whereas the two latter ones included a total of 14 investigations. Basic study characteristics of each part are separately listed in three tables (Supplemental Table 2,3,4) [16–106].

### Anti-CD20 immunotherapy

#### Baseline metabolic parameters

Among 77 included articles in the anti-CD20 immunotherapy group, 29 studies investigated the value of the metabolic baseline parameters including tumor burden indices, metabolic tumor volume (MTV), and total lesion glycolysis (TLG), as well as tumor metabolism indices, maximum of standardized uptake value (SUVmax). Significant association with progression-free survival (PFS) and overall survival (OS) was reported for both MTV and TLG in 5 articles [25, 27, 32, 104, 105]. Several articles found significant correlation between MTV [31, 52, 61, 67, 74, 76, 77, 83, 84, 92, 93, 101, 103, 106], TLG [42, 53, 61, 67, 74, 101], and SUVmax (23,35, 9,43) [43, 84, 104] with PFS. Furthermore, a significant correlation between MTV [16, 31, 76, 83, 84, 92, 93, 103], TLG [53], and SUVmax [84] with OS was reported as well. Furthermore, high levels of MTV and TLG were related to the probability of central nervous system relapse [82]. In addition, some studies indicated the combination of the baseline PET-derived metabolic parameters with patients clinical features and laboratory data [62], as well as other baseline parameters [31, 78] or interim [67, 101] or EOT response [74], can better stratify lymphoma patients outcome. Notably, a significant relation with one or two of these parameters was not essentially found in some of the mentioned investigations. One paper reported that these baseline parameters do not provide prognostic information beyond that can already be obtained by the International Prognosis Index (IPI) [16].

**Fig. 2:**
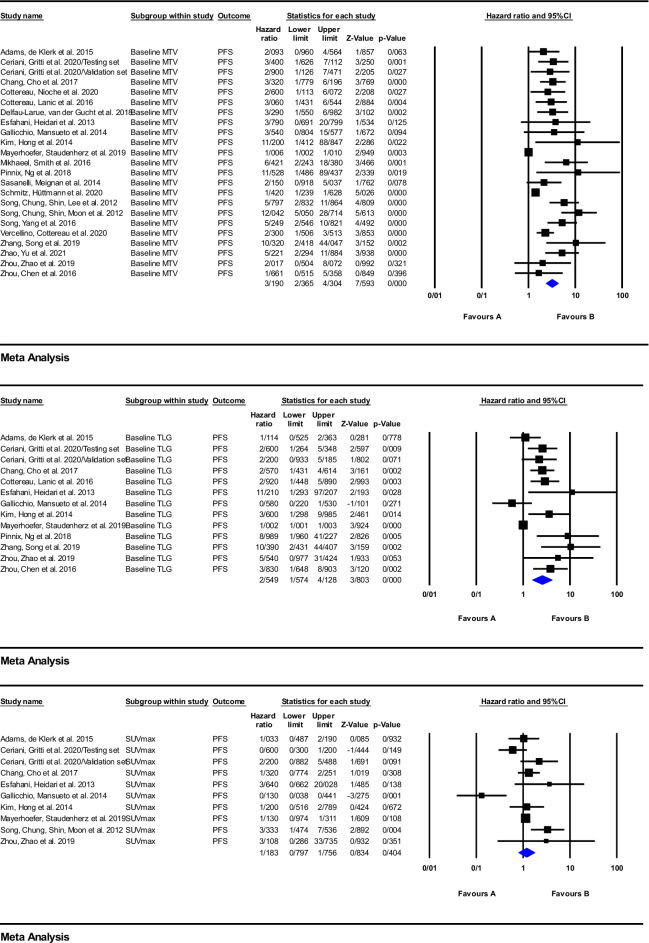
Meta-analysis of baseline parameters, MTV, TLG, and SUVmax for PFS in studies on anti-CD20 therapy

**Fig. 3:**
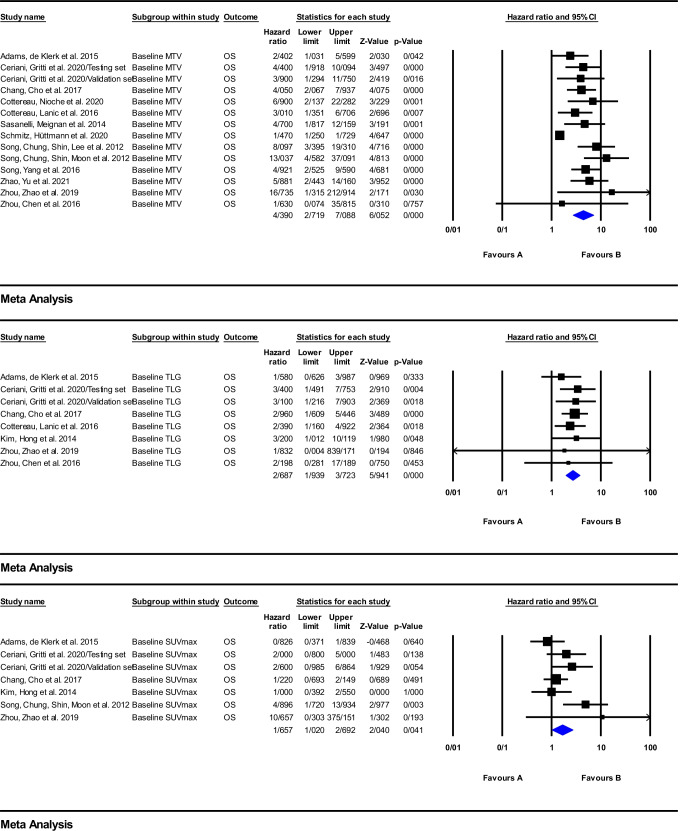
Meta-analysis of baseline parameters, MTV, TLG, and SUVmax for OS in studies on anti-CD20 therapy

According to the forest plot of the effect of baseline metabolic parameters on clinical outcome of patients treated with anti-CD20 immunotherapy, depicted in Figs. 2 and 3, the included studies reported quite heterogeneous hazard ratios from 1.1 to 16.73. On the basis of the meta-analysis calculations for PFS, one finds that the values of the pooled HRs of baseline MTV, TLG, and SUVmax were equal to 3.19 (95%CI: 2.36–4.30; *P* = 0.000), 2.54 (95%CI: 1.57–4.12; *P* = 0.000), and 1.18 (95%CI: 0.79–1.75; *P* = 0.404), respectively, while, for OS, the pooled HRs were 4.39 (95%CI: 2.71–7.08; *P* = 0.000), 2.68 (95%CI: 1.93–3.72; *P* = 0.000), and 1.65 (95%CI: 1.02–2.69; *P* = 0.041) for MTV, TLG, and SUVmax, respectively.

Bone marrow involvement based on hypermetabolic lesions in the bone marrow on baseline ^18^F-FDG PET/CT imaging is another factor which is predictive of clinical outcomes and was previously assessed in some studies [29, 30, 41, 50, 75, 81]. A poorer clinical outcome in patients with bone marrow ^18^F-FDG avid loci was reported by three investigations [29, 41, 50]. Likely, a poor outcome was associated with more than 2 sites of extra nodal involvement in baseline ^18^F-FDG PET/CT imaging [40, 41]. Another study revealed histological transformation into aggressive lymphoma types in follicular lymphoma did not have any relation with SUVmax or SUV range in baseline 18F-FDG PET/CT imaging [69].

According to the funnel plots, publication bias is probable for the pooled HR of MTV, TLG, and SUVmax for PFS as well as MTV for OS (supplemental figures).

#### Interim response assessment

**Fig. 4:**
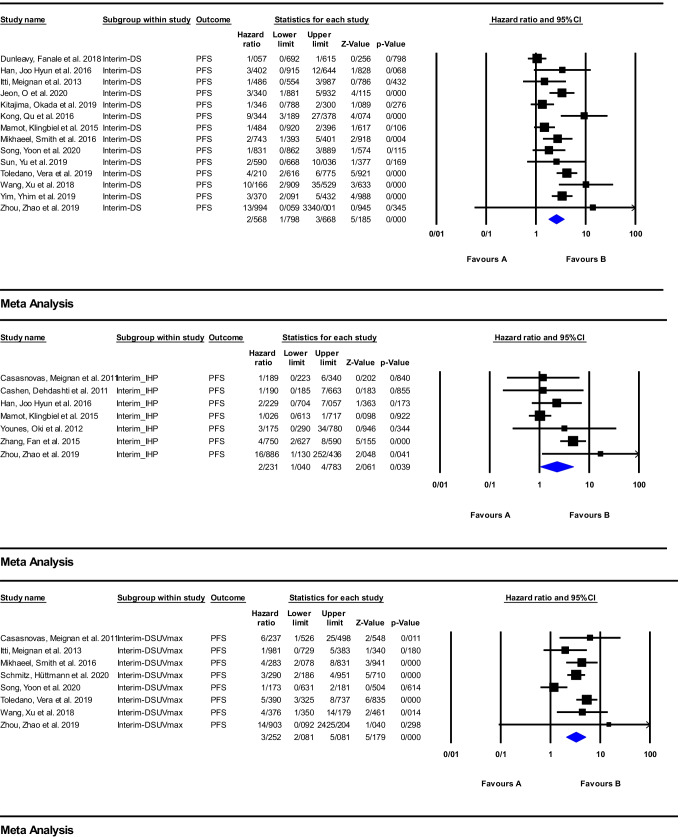
Meta-analysis of interim Response assessment using DS, IHP, and delta SUVmax for PFS in studies on anti-CD20 therapy

**Fig. 5:**
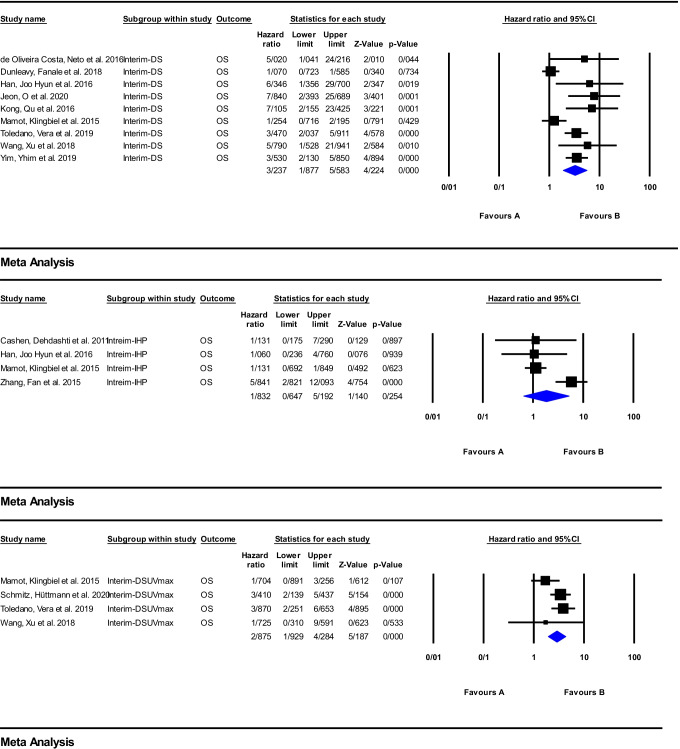
Meta-analysis of interim response assessment using DS, IHP, and delta SUVmax for OS in studies on anti-CD20 therapy

A total of 38 articles investigated response assessment via early ^18^F-FDG PET/CT imaging in the anti-CD20 immunotherapy, and of these, 19 studies were included in the meta-analysis (Supplemental Table 2) [16, 18, 21–23, 25–27, 29–35, 38–46, 48–57, 59–69, 71–78, 80–92, 96–106]. The meta-analysis was carried out on the DS, IHP, and ΔSUVmax for PFS and OS, and the obtained results are depicted as forest plots in Figs. 4 and 5. The pooled HR of the ΔSUVmax was the highest value for PFS of 3.25 (95%CI: 2.08–5.08; *P* = 0.000). However, for OS, the DS had the highest HR of 3.23 (95%CI: 1.87–5.58; *P* = 0.000). The pooled HRs of the IHP for PFS and OS were the lowest values, being 2.23 (95%CI: 1.04–4.78; *P* = 0.039) and 1.83 (95%CI: 0.64–5.19; *P* = 0.254), respectively.

Several studies have reported that interim ^18^F-FDG PET/CT could have a key role in survival prediction and early response assessment of patients with lymphoma treated with anti-CD20 therapy [21, 38, 48, 49, 55, 56, 64, 80, 99, 100, 106]. In contrast, several studies reported a poor positive predictive value in early response assessment [45, 46, 54, 60, 66, 72, 97, 104], whereby the interpretation of the ^18^F-FDG PET/CT scan is of special importance. Indeed, the results obtained through visual analysis using IHP or DS criteria have shown poor positive predictive value for recurrence compared to the semi-quantitative analysis based on SUVmax values [22, 23, 38, 45, 48]. Moreover, the combination of clinical variables and either interim-derived quantitative parameters or interim DS has been shown to improve the value of outcome prediction [56, 73, 85, 99]. In confirmation of this finding, the subgroup meta-analysis performed for interim PET scan showed the highest HR belonged to ΔSUVmax for prediction of both PFS and OS. Several studies reported a high negative predictive value for early time point ^18^F-FDG PET/CT imaging [23, 35, 68, 89]. Subsequently, some investigations reported conventional treatment intensification based on interim ^18^F-FDG PET/CT findings might had not clinical benefit and could not improve the final outcome and must be restricted to clinical trials [38, 86].

Early ^18^F-FDG PET/MRI using quantitative ^18^F-FDG PET and diffusion-weighted magnetic resonance imaging parameters has been shown to identify anti-CD20 therapy-induced changes as early as 48–72 h after treatment initiation [63, 64].

The presence of publication bias for the pooled HR of interim response assessment using DS for OS is probable (supplemental figures).

#### End of treatment (EOT) response assessment

**Fig. 6:**
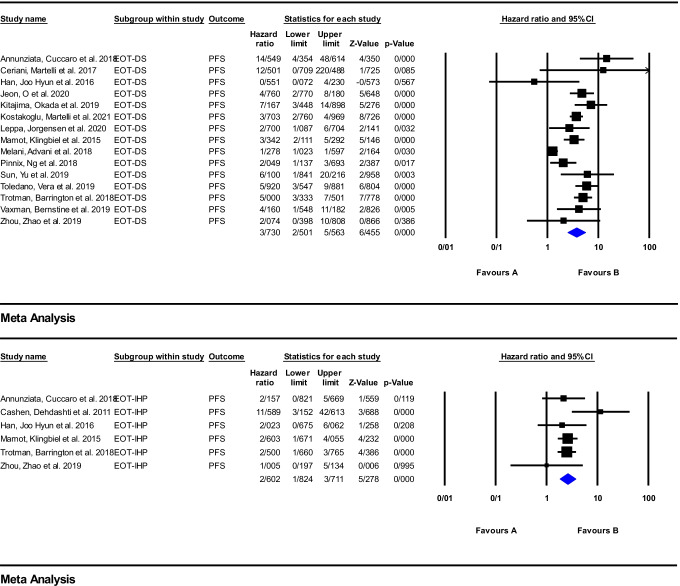
Meta-analysis of EOT response assessment using DS and IHP criteria for PFS in studies on anti-CD20 therapy

**Fig. 7:**
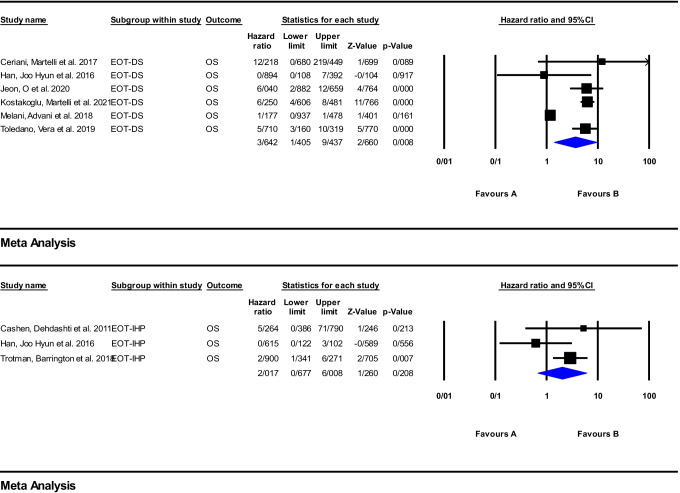
Meta-analysis of EOT Response assessment using DS and IHP criteria for OS in studies on anti-CD20 therapy

Meta-analysis was performed using 16 articles on anti-CD20 therapy. The reported HRs, for PFS, are widely ranged from 1.27 to 14.54 and 1 to 11.58 for DS and IHP, respectively. For PFS, the pooled HR of the EOT response assessment using DS and IHP was 3.73 (95%CI: 2.50–5.56; *P* = 0.000) and 2.60 (95%CI: 1.82–3.71; *P* = 0.000), respectively. For OS, this range was from 0.89 to 12.21 for DS and from 0.61 to 5.26 for IHP criteria. The pooled HR was 3.64 (95%CI: 1.40–9.43; *P* = 0.008) and 2.01 (95%CI: 0.67–6.00; *P* = 0.208), respectively. The corresponding forest plots were illustrated in Figs. 6 and 7.

For EOT ^18^F-FDG PET/CT as well, the reports on potential prognostic effect have been mixed. Several studies reported the EOT ^18^F-FDG PET/CT using different evaluation systems could have a promising role in outcome prediction [18, 23, 44, 54, 57, 60, 65, 74, 87, 88, 90, 91, 98, 104]. Aside from visual criteria, some newer quantitative parameters such as the ratio between the SUVmax of a hypermetabolic lymphoma lesion and liver SUVmax on the EOT ^18^F-FDG PET/CT have a similar prognostic power as DS in predicting of the patient clinical outcome [18, 89]. On the other hand, some investigations reported a low positive predictive value for EOT imaging, which could limit the role of ^18^F-FDG PET/CT in end of treatment response evaluation [26, 45, 46, 59, 65]. Similar to the interim imaging, a high NPV was reported on the EOT scan [46, 49, 65, 66, 68, 85, 104].

Addition of rituximab to the conventional chemotherapy resulted in reduced positive predictive value of the interim and end of treatment ^18^F-FDG PET/CT-based response assessment [45, 46]. This may in part be explained by the inflammatory reactions as a consequence of recruitment of immune cells to the tumor following rituximab treatment. Consequently, glucose consumption of the migrated immune cells might lead to a local hypermetabolism and false-positive PET results [46].

### Immune checkpoint inhibitors (ICI)

#### Baseline metabolic parameters

Unlike the large number of studies on anti-CD20 therapy, there are fewer studies on the value of ^18^F-FDG PET/CT on response assessment of ICIs in lymphoma. These results in a limitation of small sample sizes and short follow-up duration since ICI is a more recently adopted therapy. Among the nine included articles under this category, one reported that baseline MTV had no statistically significant correlation with achieving complete metabolic response after pembrolizumab therapy [17].

#### Response assessment

In the category of ICIs, early ^18^F-FDG PET/CT scans appear to be more important than end of treatment response assessment. Some studies stated that patients achieved objective response after 3 months of ICI initiation and early ^18^F-FDG PET/CT evaluation can detect responders at this time point [19, 36]. Various methods have been employed to assess early imaging including quantitative (MTV) or qualitative (DS) approaches; however, early ^18^F-FDG PET/CT findings were associated with the best overall response [19, 24, 36, 94] or patient survival [24, 28, 58, 70]. Reduction of MTV [24, 36, 94], ΔSUVmax [24, 36], decrease in TLG (92) [24], and visual analysis by DS [24, 70] was associated with the best overall response (Supplemental Table 3) [17, 19, 24, 28, 36, 58, 70, 94]. Although some of studies evaluated Lugano and LYRIC criteria, they were heterogeneous in methodology and reported results; so we could not enrolled them to meta-analysis [24, 28, 36, 70].

#### Immune-related adverse events (irAEs)

The potential irAEs related to ICI treatment include fever, headache, arthralgia, cytopenia, respiratory system involvement, gastrointestinal tract involvement, and skin rash [17, 19, 24, 58]. In one study, it was reported that the rate of irAE was higher in ICI responding patients in comparison to the non-responders; however, this finding was not significant [24]. There were no reports on irAEs leading to death in the articles reviewed [17, 24, 58].

### Cellular therapy

#### Baseline metabolic parameters

In cell therapy, among the 8 related studies, only one indicated MTV had a significant association with PFS and OS [93]. On the other hand, one article stated MTV and TLG were not associated with response to treatment and OS [95].

#### Response assessment

Early ^18^F-FDG PET/CT imaging at ~ 1 month after therapy initiation has been reported to be useful for early response assessment following cellular therapy and may have a role in guiding treatment pathway [37, 79]. For example, MTV may be an effective tool for early response assessment [79] and early suppression of glucose consumption in lymphoid organs may be associated with poor outcome [37]. Also, ^18^F-FDG PET may have a role in monitoring the immune effects after allogeneic cell transplantation [47] (Supplemental Table 4) [20, 37, 47, 79, 93, 95].

#### Immune-related adverse events (irAEs)

The most common irAEs of CAR-T cell therapy is cytokine release syndrome (CRS), and a concurrent local immune activation within a tumoral lesion that can cause a severe local inflammation which can lead to pseudo-progression. It has been reported that the baseline tumor burden was linked to CRS and pseudo-progression in treatment course [37]. It should be noted that there is a controversy in this issue and another study did not witness any relation between baseline FDG metabolic parameters and CRS [79]. Another study reported that the presence of pseudo-progression did not alter early response evaluation [58]. Moreover, another study indicated that CRS did not confound ^18^F-FDG PET/CT interpretation [47].

## Discussion

The high sensitivity of the ^18^F-FDG PET in detecting nodal and extranodal involvement has established its role in primary staging as a standard of care for all ^18^F-FDG-avid lymphomas [11]. Calculating quantitative baseline tumor burden and tumor metabolism indices following immunotherapy are common parameters used to predict prognosis in baseline ^18^F-FDG PET scans [25, 27, 32, 104, 105]. However, some studies have not found a definite correlation between one or more of these parameters with PFS or OS [37, 95]. This can, in part, be explained by heterogeneous patient characteristics, including a wide age range, different clinical stage and disease subtypes, and different software and the different ways used for definition of the marginal threshold of hypermetabolic foci [27]. Moreover, these discrepancies are obvious through the heterogeneity of reported HRs in Figs. 2 and 3. Based on the meta-analysis performed in the present work, the highest pooled HR between baseline parameters belongs to MTV with HR of 4.39 (95%CI: 2.71–7.08; *P* = 0.000] and the lowest one belongs to SUV_max_ with an HR of 1.18 (95%CI: 0.79–1.75; *P* = 0.404).

Many reports suggest response assessment via semi-quantitative/quantitative methods in lymphoma patients provides better outcome discriminators than the visual criteria regardless of the considered background reference tissue [21, 22, 38, 48]. Moreover, it has been reported that inter-observer reproducibility was higher using semi quantitative methods than the visual approaches [48]. Our findings showed the pooled HR of the early response assessment following anti-CD20 treatment using ΔSUVmax for PFS is 3.25 (95%CI: 2.08–5.08; *P* = 0.000), which is higher than the DS and IHP criteria’s pooled HRs. Pooled HR for an early evaluation concerning OS did not support this point and the pooled HR of the ΔSUVmax is lower than the DS corresponding value, 2.87 (95%CI: 1.92–4.28; *P* = 0.000] versus 3.23 (95%CI: 1.87–5.58, *P* = 0.000). On the other hand, according to some studies, it seems that visual assessment by DS is a better prognosticator than the IHP criteria [45]. Our findings in early response assessment pooled HRs also support this point. Pooled HR of the early response assessment for PFS and OS are respectively 2.56 (95%CI: 1.79–3.66; *P* = 0.000] and 3.23 (95%CI: 1.87–5.58; *P* = 0.000) for DS, which are higher than the IHP criteria’s pooled HRs. These measurements for DS and IHP were relatively similar for EOT ^18^F-FDG PET/CT. This difference can in part be explained by the more conservative nature of the IHP criteria, which uses a lower visual reference (surrounding background or mediastinal blood pool) compared to the DS, which uses liver parenchymal uptake [18]. It should be mentioned that the SUV of the reference organs may be affected by the hypermetabolic tumor burden, and this point should be considered when interpreting serial ^18^F-FDG PET/CT scans [51].

In immunotherapy with ICIs, a decrease in tumor metabolism indices as early as 8 weeks after therapy initiation occurred in responders. On the other hand, modifications of tumor burden indices occurred appreciably later. This time interval may be due to immune system reactivation and glucose consumption by tumor-infiltrated lymphocytes [24]. It seems that immune-related adverse events in immune cell therapy and ICIs have more impressive influence on 18F-FDG PET/CT scan, compared to the anti-CD20 immunotherapy. There are more anti-CD20 monoclonal antibody studies performed which allowed the analysis, while the current published studies in ICIs and cell therapy are quite heterogeneous with regard to response assessment and outcome prediction; therefore, dedicated analysis in this treatment cohort was not performed.

The number of enrolled papers on anti-CD20 monoclonal antibodies is quite different, compared to the other two categories: ICIs and cellular therapies. The latter ones have a small number of papers and do not have similar indices in all of them. This difference gives different strength to the results related to the anti-CD20 mAbs. With the support of the large number of studies on anti-CD20 mAbs, we could calculate pooled HRs, whereas meta-analysis and pooled ratio calculation were not possible for ICIs and cellular therapies. On the other hand, in the category of anti-CD20 mAbs, the depicted funnel plots showed asymmetry and probable publication bias in pooled HR of some of baseline parameters (MTV, TLG, and SUVmax for PFS and MTV for OS) as well as pooled HR of interim response assessment using DS for OS. This was the main limitation that we encountered in the present study.

## Conclusion

Our present study revealed that with anti-CD20 therapy, baseline MTV on ^18^F-FDG PET/CT has the highest HRs for both PFS and OS. In response assessment of anti-CD20 therapy, Deauville score in early and late time points has higher HRs for PFS and OS compared to the international harmonization project criteria. While early changes in ^18^F-FDG PET parameters may be predictive of response to ICIs and cell therapy treatment, further studies are required to establish the optimal response parameters following treatment of lymphoma patients.

## Supplementary Information

Below is the link to the electronic supplementary material.

Supplementary file1 (DOCX 33 KB)

Supplementary file2 (DOCX 59 KB)

Supplementary file3 (DOCX 22 KB)

Supplementary file4 (DOCX 21 KB)

Supplementary file5 (DOCX 296 KB)

## Data Availability

The datasets generated and analysed during the current study are available from the corresponding author on reasonable request.
