# Supplementary material for: The value of FDG PET/CT imaging in outcome prediction and response assessment of lymphoma patients treated with immunotherapy: a meta-analysis and systematic review
Source: Eur J Nucl Med Mol Imaging. 2022 Aug 6;49(13):4661–76. doi: 10.1007/s00259-022-05918-2 (PMC9606078; doi:10.1007/s00259-022-05918-2)
Supplement: Supplementary file 1 — Supplementary file1 (DOCX 33 KB) [file 259_2022_5918_MOESM1_ESM.docx]

Table 1. Quality control of the included articles according to the critical appraisal tool obtained from the Oxford Center for Evidence-Based Medicine.

| Authors (year) | Patient registration was at a common time point | Sufficient follow up duration applied | Outcome criteria applied in a blind fashion | Important prognostic factors adjusted |
| --- | --- | --- | --- | --- |
| Adam *et al.* (2015) *(16)* | Yes | Yes | Yes | Yes |
| [Allen](file:///C:\Users\AK\Documents\Meta\ref\Lymphoma\Pembrolizumab%20followed%20by%20AVD%20in%20untreated%20early%20unfavorable%20and%20advanced%20stage.pdf) *et al.*(2020) *(17)* | Yes | Yes | Yes | Yes |
| [Annunziata](file:///C:\Users\AK\Documents\Meta\ref\Lymphoma\FDG-PET%20CT%20at%20the%20end%20of%20immuno-chemotherapy%20in%20follicular%20lymphoma%20the%20prognostic%20role%20of%20the%20ratio%20between%20target%20lesion%20and%20liver%20SUVmax%20rPET.pdf) *et al.* (2018) *(18)* | Yes | Yes | Unclear | Yes |
| Bartlett *et al.* (2020) *(19)* | Yes | No | Yes | Yes |
| [Baudard](file:///C:\Users\AK\Documents\Meta\ref\Lymphoma\Importance%20of%20%5b18F%5dfluorodeoxyglucose-positron%20emission%20tomography%20scanning%20for%20the%20monitoring%20of%20responses%20to%20immunotherapy%20in%20follicular%20lymphoma.pdf) *et al.* (2007) *(20)* | Yes | No | Unclear | Yes |
| Casasnovas *et al.* (2017) *(21)* | Yes | Yes | Yes | Yes |
| [Casasnovas](file:///C:\Users\AK\Documents\Meta\ref\Lymphoma\SUVmax_reduction_improves_early_prognosis_value_of.pdf) *et al.* (2011) *(22)* | Yes | Yes | Yes | Yes |
| Cashen *et al.* (2011) *(23)* | Yes | Yes | Yes | Yes |
| Castello *et al.* (2019) *(24)* | Yes | Yes | Unclear | Yes |
| Ceriani *et al.* (2020) *(25)* | Yes | Yes | Yes | Yes |
| [Ceriani](file:///C:\Users\AK\AppData\Roaming\Microsoft\PET%20CT%20assessment%20after%20immunochemotherapy%20and%20irradiation%20using%20the%20Lugano.pdf) *et al.* (2017) *(26)* | Yes | Yes | Yes | Yes |
| [Chang](file:///C:\Users\AK\Documents\Meta\ref\Lymphoma\Prognostic%20significance%20of%20total%20metabolic%20tumor%20volume.pdf) *et al.* (2017) *(27)* | Yes | Yes | Yes | Yes |
| Chen *et al.* (2019)*(28)* | Yes | Yes | Yes | Yes |
| Chen *et al.* (2018) *(29)* | Yes | Yes | Yes | Yes |
| Chen-Liang *et al.* (2017) *(30)* | Yes | Yes | Unclear | Yes |
| [Cottereau](file:///C:\Users\AK\Documents\Meta\ref\Lymphoma\18F-FDG%20PET%20Dissemination%20Features%20in%20Diffuse%20Large%20B-Cell.pdf) *et al.* (2020) *(31)* | Yes | Yes | Yes | Yes |
| [Cottereau](file:///C:\Users\AK\Documents\Meta\ref\Lymphoma\Molecular%20Profile%20and%20FDG-PET%20CT%20Total.pdf) *et al.* (2016) *(32)* | Yes | Yes | Yes | Yes |
| Cui *et al.* (2018) *(33)* | Yes | No | Yes | Unclear |
| Delfau-Larue *et al.* (2018) *(34)* | Yes | Yes | Yes | Yes |
| de Oliveira Costa *et al.* (2016) *(35)* | Yes | Yes | Yes | Yes |
| Dercle *et al.* (2018) *(36)* | Yes | No | Yes | Yes |
| Derlin *et al.* (2021) *(37)* | Yes | No | Unclear | Yes |
| [Duhrsen](file:///C:\Users\AK\Documents\Meta\ref\Lymphoma\Positron%20Emission%20Tomography–Guided%20Therapy%20of%20agressive.pdf) *et al.* (2018) *(38)* | Yes | Yes | Yes | Yes |
| [Dunleavy](file:///C:\Users\AK\Documents\Meta\ref\Lymphoma\A%20Prospective%20Multicenter%20Phase%202%20Study%20of%20Dose-AdjustedEPOCH-R%20in%20Untreated%20MYC-Rearranged%20Aggressive%20B-cell.pdf) *et al.* (2018) *(39)* | Yes | Yes | Unclear | Yes |
| [El-Galaly](file:///C:\Users\AK\AppData\Roaming\Microsoft\The%20number%20of%20extranodal%20sites%20assessed%20by%20PET%20CT%20scan.pdf) *et al.* (2017) *(40)* | Yes | Yes | Unclear | Yes |
| [El-Galaly](file:///C:\Users\AK\AppData\Roaming\Microsoft\Outcome%20prediction%20by%20extranodal%20involvement,%20IPI,%20R-IPI,.pdf) *et al.* (2015) *(41)* | Yes | Yes | Yes | Yes |
| [Esfahani](file:///C:\Users\AK\Documents\Meta\ref\Lymphoma\Baseline%20total%20lesion%20glycolysis%20measured%20with%2018F-FDG.pdf) *et al.* (2013) *(42)* | Yes | Yes | Yes | Yes |
| [Gallicchio](file:///C:\Users\AK\Documents\Meta\ref\Lymphoma\F-18%20FDG%20PET%20CT%20quantization%20parameters%20as%20predictors%20of.pdf) *et al.* (2014) *(43)* | Yes | Yes | Yes | Yes |
| Giulino-Roth *et al.* (2017) *(44)* | Yes | Yes | Unclear | Yes |
| Han *et al.* (2016) *(45)* | Yes | Yes | Yes | Yes |
| Han *et al.* (2009) *(46)* | Yes | Yes | Yes | Yes |
| [Hart](file:///C:\Users\AK\AppData\Roaming\Microsoft\Use%20of18F-FDG%20positron%20emission%20tomography%20followingallogeneic%20transplantation%20to%20guide%20adoptive%20immunotherapywith%20donor%20lymphocyte%20infusions.pdf) *et al.* (2005) *(47)* | Yes | No | Unclear | Yes |
| [Itti](file:///C:\Users\AK\Documents\Meta\ref\Lymphoma\An%20international%20confirmatory%20study%20of%20the%20prognostic%20value.pdf) *et al.* (2013) *(48)* | Yes | Yes | Yes | Yes |
| Jeon *et al.* (2020) *(49)* | Yes | Yes | Unclear | Yes |
| [Khan](file:///C:\Users\AK\Documents\Meta\ref\Lymphoma\PET-CT%20staging%20of%20DLBCL%20accurately%20identifies%20and%20provides%20newinsight%20into%20the%20clinical%20significance%20of%20bone%20marrow%20involvement.pdf) *et al.* (2013) *(50)* | Yes | Yes | Yes | Yes |
| Kim *et al.* (2016) *(51)* | Yes | No | Unclear | Yes |
| Kim *et al.* (2014) *(52)* | Yes | Yes | Yes | Yes |
| [Kim](file:///C:\Users\AK\Documents\Meta\ref\Lymphoma\Total%20Lesion%20Glycolysis%20in%20Positron%20Emission%20Tomography%20Is%20a.pdf) *et al.* (2013) *(53)* | Yes | Yes | Unclear | Yes |
| [Kitajima](file:///C:\Users\AK\Documents\Meta\ref\Lymphoma\Predictive%20value%20of%20interim%20FDG-PET-CT%20findings%20in%20patients%20with.pdf) *et al.* (2019) *(54)* | Yes | Yes | Yes | Yes |
| [Kocurek](file:///C:\Users\AK\Documents\Meta\ref\Lymphoma\Primary%20mediastinal%20B%20cell%20lymphoma%20%20%20metabolic%20and%20anatomical%20features%20in%2018FDG%20PET%20CT%20and%20response%20to%20therapy.pdf) *et al.* (2016) *(55)* | Yes | Yes | Unclear | Yes |
| Kong *et al.* (2016) *(56)* | Yes | Yes | Yes | Yes |
| Kostakoglu *et al.* (2021) *(57)* | Yes | Yes | Yes | Yes |
| Lepik *et al.*(2020) *(58)* | Yes | Yes | Unclear | Yes |
| Leppä *et al.* (2020) *(59)* | Yes | Yes | Unclear | Yes |
| [Mamot](file:///C:\Users\AK\Documents\Meta\ref\Lymphoma\Final%20Results%20of%20a%20Prospective%20Evaluation%20of%20the%20Predictive.pdf) *et al.* (2015) *(60)* | Yes | Yes | Unclear | Yes |
| [Mayerhoefer](file:///C:\Users\AK\Documents\Meta\ref\Lymphoma\Pre-Therapeutic%20Total%20Lesion%20Glycolysis%20on%20%5b18F%5dFDG-PET%20Enables%20Prognostication%20of%202%20Year%20Progression%20Free%20Survival%20in%20MALT%20Lymphoma%20Patients%20Treated%20with%20CD20%20Antibody%20Based%20Immunotherapy.pdf) *et al.* (2019) *(61)* | Yes | Yes | Yes | Yes |
| [Mayerhoefer](file:///C:\Users\AK\Documents\Meta\ref\Lymphoma\Radiomic%20features%20of%20glucose%20metabolism%20enable%20prediction.pdf) *et al.* (2019) *(62)* | Yes | Yes | Unclear | Yes |
| [Mayerhoefer](file:///C:\Users\AK\Documents\Meta\ref\Lymphoma\Ultra-early%20response%20assessment%20in%20lymphoma%20treatment%2018F%20FDG%20PET%20MR%20captures%20changes%20in%20glucose%20metabolism%20and%20cell%20density%20within%20the%20first%2072%20hours%20of%20treatment.pdf) *et al.* (2018) *(63)* | Yes | No | Unclear | Unclear |
| [Mayerhoefer](file:///C:\Users\AK\Documents\Meta\ref\Lymphoma\Can%20Interim%2018F-FDG%20PET%20or%20Diffusion-Weighted%20MRI%20Predict%20End-of-Treatment%20Outcome%20in%20FDG-Avid%20MALT%20Lymphoma%20After%20Rituximab-Based%20Therapy.pdf) *et al.* (2016) *(64)* | Yes | No | Unclear | Unclear |
| [Melani](file:///C:\Users\AK\Documents\Meta\ref\Lymphoma\End-of-treatment%20and%20serial%20PET%20imaging%20in%20primary%20mediastinal%20B-cell%20lymphoma%20following%20dose-adjusted%20EPOCH-R%20a%20paradigm%20shift%20in%20clinical%20decision%20making.pdf) *et al.* (2018) *(65)* | Yes | Yes | Yes | Yes |
| Micallef *et al.* (2011) *(66)* | Yes | Yes | Yes | Yes |
| [Mikhaeel](file:///C:\Users\AK\Documents\Meta\ref\Lymphoma\Combination%20of%20baseline%20metabolic%20tumour%20volume%20and%20early.pdf) *et al.* (2016) *(67)* | Yes | Yes | Yes | Yes |
| Minamimoto *et al.* (2016) *(68)* | Yes | No | Yes | Yes |
| [Mir](file:///C:\Users\AK\Documents\Meta\ref\Lymphoma\Baseline%20SUVmax%20did%20not%20predict%20histological%20transformation%20in%20follicular%20lymphoma%20in%20the%20phase%203%20GALLIUM%20study.pdf) *et al.* (2020) *(69)* | Yes | No | Yes | Yes |
| Mokrane *et al.* (2020) *(70)* | Yes | Yes | Yes | Yes |
| Morschhauser *et al.* (2021) *(71)* | Yes | Yes | Yes | Yes |
| Moskowitz *et al.* (2010) *(72)* | Yes | Yes | Unclear | Yes |
| OÑATE‑OCAÑA *et al.* *(73)* | Yes | Yes | Unclear | Yes |
| Pinnix *et al.* (2018) *(74)* | Yes | Yes | Yes | Yes |
| [Rutherford](file:///C:\Users\AK\Documents\Meta\ref\Lymphoma\Impact%20of%20bone%20marrow%20biopsy%20on%20response%20assessment%20in.pdf) *et al.* (2020) *(75)* | Yes | Yes | Yes | Yes |
| [Sasanelli](file:///C:\Users\AK\Documents\Meta\ref\Lymphoma\Pretherapy%20metabolic%20tumour%20volume%20is%20an%20independent%20predictor.pdf) *et al.* (2014) *(76)* | Yes | Yes | Unclear | Yes |
| [Schmitz](file:///C:\Users\AK\Documents\Meta\ref\Lymphoma\Dynamic%20risk%20assessment%20based%20on%20positron%20emissiontomography%20scanning%20in%20diffuse%20large%20B-cell%20lymphomaPost-hoc%20analysis%20from%20the%20PETAL%20trial.pdf) *et al.* (2020) *(77)* | Yes | Yes | Yes | Yes |
| Senjo *et al.* (2020) *(78)* | Yes | Yes | Yes | Yes |
| [Shah](file:///C:\Users\AK\Documents\Meta\ref\Lymphoma\Early%20positron%20emission%20tomographycomputed%20tomography%20as%20a.pdf) *et al.* (2018) *(79)* | Yes | No | Unclear | Unclear |
| Song *et al.* (2020) *(80)* | Yes | Yes | Yes | Yes |
| [Song](file:///C:\Users\AK\Documents\Meta\ref\Lymphoma\High%20total%20metabolic%20tumor%20volume%20in%20PET%20CT%20predicts%20worse.pdf) *et al.* (2016) *(81)* | Yes | Yes | Unclear | Yes |
| Song *et al.* (2015) *(82)* | Yes | Yes | Unclear | Yes |
| [Song](file:///C:\Users\AK\Documents\Meta\ref\Lymphoma\Clinical%20significance%20of%20metabolic%20tumor%20volume%20by%20PETCTin%20stages%20II%20and%20III%20of%20diffuse%20large%20B%20cell%20lymphomawithout%20extranodal%20site%20involvement.pdf) *et al.* (2012) *(83)* | Yes | Yes | Unclear | Yes |
| [Song](file:///C:\Users\AK\Documents\Meta\ref\Lymphoma\Prognostic%20value%20of%20metabolic%20tumor%20volume%20on.pdf) *et al.* (2012) *(84)* | Yes | Yes | Unclear | Yes |
| [Sun](file:///C:\Users\AK\Documents\Meta\ref\Lymphoma\Risk%20Stratification%20Of%20Diffuse%20Large%20B-Cell%20Lymphoma%20With%20Interim%20PET%20CT%20By%20Combining%20Deauville%20Scores%20And%20International%20Prognostic%20Index.pdf) *et al.* (2019) *(85)* | Yes | Yes | Unclear | Yes |
| Swinnen *et al.* (2015) *(86)* | Yes | Yes | Yes | Yes |
| Takasaki *et al.* (2015) *(87)* | Yes | Yes | Unclear | Yes |
| Tateishi *et al.* (2015) *(88)* | Yes | Yes | Yes | Yes |
| [Toledano](file:///C:\Users\AK\Documents\Meta\ref\Lymphoma\Comparison%20of%20therapeutic%20evaluation%20criteria.pdf) *et al.* (2019) *(89)* | Yes | Yes | Unclear | Yes |
| [Trotman](file:///C:\Users\AK\Documents\Meta\ref\Lymphoma\Prognostic%20value%20of%20end%20of%20induction%20PET%20response%20after.pdf) *et al.* (2018) *(90)* | Yes | Yes | Yes | Yes |
| [Vaxman](file:///C:\Users\AK\Documents\Meta\ref\Lymphoma\FDG%20PET%20CT%20as%20a%20diagnostic%20and%20prognostic%20tool%20for%20the%20evaluation%20of%20marginal%20zone%20lymphoma.pdf) *et al.* (2019) *(91)* | Yes | Yes | Yes | Yes |
| Vercellino *et al.* (2020) *(92)* | Yes | Yes | Yes | Yes |
| Vercellino *et al.* (2020) *(93)* | Yes | Yes | Yes | Yes |
| Voltin *et al.* (2019) *(94)* | Yes | No | Yes | Yes |
| Wang *et al.* (2019) *(95)* | Yes | Yes | Yes | Yes |
| Wang *et al.* (2018) *(96)* | Yes | Yes | Unclear | Yes |
| [Wei](file:///C:\Users\AK\Documents\Meta\ref\Lymphoma\A%20single%20center%20experience%20rituximab%20plus%20cladribine%20is%20an.pdf) *et al.* (2017) *(97)* | Yes | Yes | Unclear | Yes |
| Wong-Sefidan *et al.* (2017) *(98)* | Yes | Yes | Yes | Yes |
| [Yim](file:///C:\Users\AK\AppData\Roaming\Microsoft\Early%20risk%20stratification%20for%20diffuse%20large%20B-cell%20lymphoma%20integrating.pdf) *et al.* (2019) *(99)* | Yes | Yes | Yes | Yes |
| Younes *et al.* (2012) *(100)* | Yes | Yes | Unclear | Yes |
| Zhang *et al.* (2019) *(101)* | Yes | Yes | Yes | Yes |
| Zhang *et al.* (2015) *(102)* | Yes | Yes | Yes | Yes |
| Zhao *et al.* (2021) *(103)* | Yes | Yes | Unclear | Yes |
| [Zhou](file:///C:\Users\AK\Documents\Meta\ref\Lymphoma\Prognostic%20values%20of%20baseline,%20interim%20and%20end-of%20therapy%2018F-FDG%20PET%20CT%20in%20patients%20with%20follicular%20lymphoma.pdf) *et al.* (2019) *(104)* | Yes | Yes | Unclear | Yes |
| [Zhou](file:///C:\Users\AK\Documents\Meta\ref\Lymphoma\Prognostic%20value%20of%20total%20lesion%20glycolysis%20of%20baseline.pdf) *et al.* (2016) *(105)* | Yes | Yes | Yes | Yes |
| Zinzani *et al.* (2011) *(106)* | Yes | Yes | Unclear | Yes |
