# Supplementary material for: The value of FDG PET/CT imaging in outcome prediction and response assessment of lymphoma patients treated with immunotherapy: a meta-analysis and systematic review
Source: Eur J Nucl Med Mol Imaging. 2022 Aug 6;49(13):4661–76. doi: 10.1007/s00259-022-05918-2 (PMC9606078; doi:10.1007/s00259-022-05918-2)
Supplement: Supplementary file 2 — Supplementary file2 (DOCX 59 KB) [file 259_2022_5918_MOESM2_ESM.docx]

Table 2 Basic characteristics of the 77 included articles in lymphoma patients treated with Anti-CD-20 immunotherapy.

| Authors  (year) | Lymphoma type, sample size | Treatment | Imaging modality & intervals | Response assessment method | Clinical outcome | Conclusion |
| --- | --- | --- | --- | --- | --- | --- |
| [Adam](file:///C:\Users\AK\Documents\Meta\ref\Lymphoma\Prognostic%20superiority%20of%20the%20National%20Comprehensive%20Cancer%20Network%20International.pdf) *et al.* *^a^* (2015) *(16)* | NHL: 73  Newly diagnosed DLBCL | Anti-CD20  (Rituximab) | - 18F-FDG PET/CT × 1:   Baseline | - Quantitative (MTV, TLG & SUVmax) | OS & PFS | - In DLBCL, baseline SUVmax, MTV, & TLG did not provide prognostic information beyond that could already be obtained by the NCCN-IPI. - Significant association with OS was observed only for MTV in univariate analysis. |
| [Annunziata](file:///C:\Users\AK\Documents\Meta\ref\Lymphoma\FDG-PET%20CT%20at%20the%20end%20of%20immuno-chemotherapy%20in%20follicular%20lymphoma%20the%20prognostic%20role%20of%20the%20ratio%20between%20target%20lesion%20and%20liver%20SUVmax%20rPET.pdf) *et al. ^c^*  (2018) *(18)* | NHL: 89 | Anti-CD20  (Rituximab) | - 18F-FDG PET/CT × 1:   EOT | - Quantitative (SUVmax, Target SUVmax/liver SUVmax) - DS - IHP | PFS | - All EOT PET data (IHP, DS & Target SUVmax/liver SUVmax) showed significant association with PFS at univariate and multivariate analysis. |
| [Casasnovas](file:///C:\Users\AK\Documents\Meta\ref\Lymphoma\FDG-PET–driven%20consolidation%20strategy%20in%20diffuse%20large%20B-cell%20lymphoma.pdf) *et al.*  (2017) *(21)* | NHL: 222  DLBCL | Anti-CD20  (Rituximab) | - 18F-FDG PET/CT × 3:   Baseline  Interim (2 cycles)  Interim (4 cycles) | - Quantitative (ΔSUVmax) - IHP | OS & PFS | - ΔSUVmax >70% was related with a better outcome. |
| [Casasnovas](file:///C:\Users\AK\Documents\Meta\ref\Lymphoma\SUVmax_reduction_improves_early_prognosis_value_of.pdf) *et al. ^b^*  (2011) *(22)* | NHL: 130  DLBCL | Anti-CD20  (Rituximab) | - 18F-FDG PET/CT × 3:   Baseline  Interim (2 cycles)  Interim (4 cycles) | - Quantitative (ΔSUVmax) - IHP | OS & PFS | - ΔSUVmax analysis of interim PET scans was feasible for high risk DLBCL and was a better outcome predictor than visual analysis. |
| [Cashen *et al.*](file:///C:\Users\AK\Documents\Meta\ref\Lymphoma\new\18F-FDG%20PET%20CT%20for%20Early%20Response%20Assessment%20in%20Diffuse.pdf) *^b, c^*  (2011) *(23)* | NHL: 50  Ad DLBCL | Anti-CD20  (Rituximab) | - 18F-FDG PET/CT × 2:   Interim (2/3 cycles)  EOT | - Quantitative (SUVmax) - IHP | OS & PFS | - According to IHP criteria, early response assessment with PET/CT had have a high NPV but low PPV in patients with advanced-stage DLBCL. |
| [Ceriani *et al.*](file:///C:\Users\AK\Documents\Meta\ref\Lymphoma\new\SAKK3807%20study%20integration%20of%20baseline%20metabolic%20heterogeneity%20and.pdf) *^a^*  (2020) *(25)* | NHL: 141  DLBCL | Anti-CD20  (Rituximab) | - 18F-FDG PET/CT × 3:   Baseline  Interim  EOT | - Quantitative (MTV, TLG, SUVmax & MH) | OS & PFS | - Elevated MTV and TLG baseline parameters were significantly associated with worse PFS and OS, whereas SUVmax and MH had no effect on patient’s outcome. |
| [Ceriani *et al.*](file:///C:\Users\AK\Documents\Meta\ref\Lymphoma\PET%20CT%20assessment%20after%20immunochemotherapy%20and%20irradiation%20using%20the%20Lugano.pdf) *^c^*  (2017) *(26)* | NHL: 125  PMBCL | Anti-CD20  (Rituximab) | - 18F-FDG PET/CT × 1:   EOT (after radiotherapy) | - Lugano - DS | OS & PFS | - All the patients obtaining a CMR defined by a DS≤3 remained progression-free at 5 years, confirming the excellent NPV of the Lugano criteria in PMBCL patients. - The few patients with DS4 also had an excellent outcome, suggesting that they did not necessarily require additional therapy, since the residual FDG uptake may not reflect persistent lymphoma. |
| [Chang](file:///C:\Users\AK\Documents\Meta\ref\Lymphoma\Prognostic%20significance%20of%20total%20metabolic%20tumor%20volume.pdf) *et al. ^a^*  (2017) *(27)* | NHL: 118  DLBCL | Anti-CD20  (Rituximab) | - 18F-FDG PET/CT × 1:   Baseline | - Quantitative (MTV, TLG, SUVmax & SUVmean) | OS & PFS | - MTV and TLG baseline parameters significantly associated with PFS and OS, whereas SUVmax had no signification correlation with outcomes. |
| [Chen *et al.*](file:///C:\Users\AK\Documents\Meta\ref\Lymphoma\new\Prognostic%20Value%20of%20Bone%20Marrow%20FDG%20Uptake%20Pattern.pdf)  (2018) *(29)* | NHL: 193  DLBCL | Anti-CD20  (RITUXIMAB) | - 18F-FDG PET/CT × 1:   Baseline | - Visually (Bone Marrow involvement) | OS & PFS | - PET/CT-directed bone marrow patterns were meaningful in predicting prognosis of newly diagnosed DLBCL patients. Focal BM uptake pattern was an independent predictor for PFS. |
| [Chen-Liang *et al.*](file:///C:\Users\AK\Documents\Meta\ref\Lymphoma\new\Bone%20marrow%20biopsy%20superiority%20over%20PET%20CT%20in%20predicting.pdf)  (2017) *(30)* | NHL: 268  DLBCL | Anti-CD20  (Rituximab) | - 18F-FDG PET/CT × 1:   Baseline | - Visually (Bone Marrow involvement) - Quantitative (SUVmax of bone marrow) | OS & PFS | - Bone marrow involvement in FDG PET/CT could not independently predict a shorter PFS or OS. |
| [Cottereau](file:///C:\Users\AK\Documents\Meta\ref\Lymphoma\18F-FDG%20PET%20Dissemination%20Features%20in%20Diffuse%20Large%20B-Cell.pdf) *et al. ^a^*  (2020) *(31)* | NHL: 95  Ad DLBCL | Anti-CD20  (Rituximab) | - 18F-FDG PET/CT × 1:   Baseline | - Quantitative (MTV, SUVmax & TLG) | OS & PFS | - Combining MTV with a parameter reflecting the tumor dissemination improved risk stratification at staging of DLBCL patients. - MTV baseline parameter significantly correlated with PFS and OS. |
| [Cottereau](file:///C:\Users\AK\Documents\Meta\ref\Lymphoma\Molecular%20Profile%20and%20FDG-PET%20CT%20Total.pdf) *et al. ^a^*  (2016) *(32)* | NHL: 81  Newly diagnosed DLBCL | Anti-CD20  (Rituximab) | - 18F-FDG PET/CT × 1:   Baseline | - Quantitative (MTV, TLG, SUVmax & SUVmean) | OS & PFS | - MTV and TLG baseline parameters were prognostic factors of both PFS and OS, whereas SUVmax had not correlation with PFS or OS. |
| [Cui](C:\\Users\\AK\\Documents\\Meta\\ref\\Lymphoma\\Circulating cell-free miR-494 and miR-21 are disease response.pdf) *[et al.](C:\\Users\\AK\\Documents\\Meta\\ref\\Lymphoma\\Circulating cell-free miR-494 and miR-21 are disease response.pdf)*  (2018) *(33)* | NHL: 99  DLBCL | Anti-CD20  (Rituximab) | - 18F-FDG PET/CT × 2:   Baseline  Interim | - DS | NA | - Circulating RNAs were disease response biomarkers with differential response that could stratify by interim FDG PET/CT in patients with DLBCL. |
| [Delfau-Larue *et al.*](file:///C:\Users\AK\Documents\Meta\ref\Lymphoma\new\Total%20metabolic%20tumor%20volume,%20circulating%20tumor%20cells,%20cell-free.pdf) *^a^* *(34)*  (2018) | NHL: 133  FL | Anti-CD20  (Rituximab) | - 18F-FDG PET/CT × 1:   Baseline | - Quantitative (MTV) | PFS | - PFS was shorter for patients with high baseline TMTV. |
| [de Oliveira Costa *et al.*](file:///C:\Users\AK\Documents\Meta\ref\Lymphoma\new\Interim%20fluorine-18%20fluorodeoxyglucose%20PET-computed.pdf) *^b^*  (2016) *(35)* | NHL: 111  DLBCL | Anti-CD20  (Rituximab) | - 18F-FDG PET/CT × 3:   Baseline  Interim (2 cycles)  EOT | - Visually - DS | OS & PFS | - Interim FDG PET/CT was an independent predictor for OS. |
| [Duhrsen](file:///C:\Users\AK\Documents\Meta\ref\Lymphoma\Positron%20Emission%20Tomography–Guided%20Therapy%20of%20agressive.pdf) *et al.*  (2018) *(38)* | NHL: 862  aggressive B/T cell lymphoma | Anti-CD20  (Rituximab) | - 18F-FDG PET/CT × 2:   Interim (2 cycles)  EOT | - Quantitative (ΔSUVmax) - DS | OS & PFS | - Interim FDG PET/CT predicted survival in patients with aggressive lymphomas. - Treatment intensification based on FDG PET did not improve outcome. |
| [Dunleavy](file:///C:\Users\AK\Documents\Meta\ref\Lymphoma\A%20Prospective%20Multicenter%20Phase%202%20Study%20of%20Dose-AdjustedEPOCH-R%20in%20Untreated%20MYC-Rearranged%20Aggressive%20B-cell.pdf) *et al.* *^b^*  (2018) *(39)* | NHL: 53  HGBCL | Anti-CD20  (Rituximab) | - 18F-FDG PET/CT × 1:   Interim (2 cycles) | - DS | OS & PFS | - Although patients with a positive interim FDG PET scan had a worse outcome, over 60% of them nevertheless achieved durable remissions. |
| [El-Galaly](C:\\Users\\AK\\Documents\\Meta\\ref\\Lymphoma\\The number of extranodal sites assessed by PET CT scan.pdf) *[et al.](C:\\Users\\AK\\Documents\\Meta\\ref\\Lymphoma\\The number of extranodal sites assessed by PET CT scan.pdf)*  (2017) *(40)* | NHL: 1532  DLBCL | Anti-CD20  (Rituximab) | - 18F-FDG PET/CT × 1:   Baseline | - Visually (Extranodal involvement) | PFS | - Patients with FDG uptake in more than 2 extranodal sites should be considered for baseline CNS involvement screening. |
| [El-Galaly](C:\\Users\\AK\\Documents\\Meta\\ref\\Lymphoma\\Outcome prediction by extranodal involvement, IPI, R-IPI,.pdf) *[et al.](C:\\Users\\AK\\Documents\\Meta\\ref\\Lymphoma\\Outcome prediction by extranodal involvement, IPI, R-IPI,.pdf)*  (2015) *(41)* | NHL: 443  DLBCL | Anti-CD20  (Rituximab) | - 18F-FDG PET/CT × 2:   Baseline  EOT | - Visually (Extranodal involvement) | OS & PFS | - Bone/BM involvement was the most commonly involved extranodal site identified by PET/CT and was associated with worse PFS and OS. - Involvement of more than 2 extranodal sites is associated with a dismal outcome. - The presence of any extranodal disease was associated with a worse PFS in univariate and multivariate analyses. |
| [Esfahani](file:///C:\Users\AK\Documents\Meta\ref\Lymphoma\Baseline%20total%20lesion%20glycolysis%20measured%20with%2018F-FDG.pdf) *et al.* *^a^*  (2013) *(42)* | NHL: 20  DLBCL | Anti-CD20  (Rituximab) | - 18F-FDG PET/CT × 3:   Baseline  Interim (2 cycles)  EOT (after completion) | - Quantitative (MTV, TLG, SUVmax & SUVmean) | PFS | - The TLG was the only discriminator of recurrence at baseline. - Among the interim FDG PET/CT parameters, SUVmean, SUVmax, and TLG could help predict PFS. |
| [Gallicchio](file:///C:\Users\AK\Documents\Meta\ref\Lymphoma\F-18%20FDG%20PET%20CT%20quantization%20parameters%20as%20predictors%20of.pdf) *et al.* *^a^*  (2014) *(43)* | NHL: 52  Newly diagnosed DLBCL | Anti-CD20  (Rituximab) | - 18F-FDG PET/CT × 1:   Baseline | - Quantitative (MTV, TLG & SUVmax) | PFS | - The SUVmax rather than MTV and TLG remains the only predictor for PFS in DLBCL patients. |
| [Giulino-Roth *et al.*](file:///C:\Users\AK\Documents\Meta\ref\Lymphoma\new\bin\Outcomes%20of%20Adults%20and%20Children%20with%20Primary%20Mediastinal%20B.pdf)  (2017) *(44)* | NHL: 156  PMBCL | Anti-CD20  (Rituximab) | - 18F-FDG PET/CT × 1:   EOT | - DS | OS & PFS | - Patients with a positive EOT FDG PET scan have an inferior outcome. |
| [Han](C:\\Users\\AK\\Documents\\Meta\\ref\\Lymphoma\\new\\FDG PETCT response in diffuse large B-cell.pdf) *[et al.](C:\\Users\\AK\\Documents\\Meta\\ref\\Lymphoma\\new\\FDG PETCT response in diffuse large B-cell.pdf) ^[b, c](C:\\Users\\AK\\Documents\\Meta\\ref\\Lymphoma\\new\\FDG PETCT response in diffuse large B-cell.pdf)^*  (2016) *(45)* | NHL: 59  DLBCL | Anti-CD20  (Rituximab) | - 18F-FDG PET/CT × 3:   Baseline  Interim (3 cycles)  EOT (after completion) | - IHP - DS | OS & PFS | - Interim PET/CT results by a more experienced reader using DS were significant factor for OS. - Positive interim PET/CT and negative PET/CT scans had significantly different OS. EOT PET/CT applying the DS was not significantly associated with PFS and OS. |
| [Han *et al.*](file:///C:\Users\AK\Documents\Meta\ref\Lymphoma\new\High%20incidence%20of%20false-positive%20PET%20scans%20in%20patients.pdf)  (2009) *(46)* | NHL: 51  DLBCL & MCL | Anti-CD20  (Rituximab) | - 18F-FDG PET/CT × 3:   Baseline  Interim (2-4 cycles)  Or EOT (after completion) | - Visually | OS & PFS | - Addition of rituximab resulted in reduced PPV and sensitivity of interim and EOT FDG PET in patients with aggressive B-cell NHL. |
| [Itti](file:///C:\Users\AK\Documents\Meta\ref\Lymphoma\An%20international%20confirmatory%20study%20of%20the%20prognostic%20value.pdf) *et al.* *^b^*  (2013) *(48)* | NHL: 114  DLBCL | Anti-CD20  (Rituximab) | - 18F-FDG PET/CT × 2:   Baseline  Interim (2 cycles) | - Quantitative (ΔSUVmax) - DS | OS & PFS | - Although the DS was valid for assessing the prognostic value of early PET/CT in DLBCL, the ΔSUVmax led to better performance and interobserver reproducibility and should be preferred when a baseline FDG PET scan is available. |
| [Jeon *et al.*](file:///C:\Users\AK\Documents\Meta\ref\Lymphoma\new\Prognostic%20impact%20of%20interim%20positron%20emission%20tomography%20in.pdf) *^b, c^*  (2020) *(49)* | NHL: 89  MCL | Anti-CD20  (Rituximab) | - 18F-FDG PET/CT × 3:   baseline  Interim (3 cycles)  EOT | - DS | OS & PFS | - Interim PET was an independent predictor of survival in MCL patients. - Early metabolic responders showed better OS and PFS than delayed metabolic responders and non-metabolic responders. |
| [Khan](file:///C:\Users\AK\Documents\Meta\ref\Lymphoma\PET-CT%20staging%20of%20DLBCL%20accurately%20identifies%20and%20provides%20newinsight%20into%20the%20clinical%20significance%20of%20bone%20marrow%20involvement.pdf) *et al.*  (2013) *(50)* | NHL: 130  DLBCL | Anti-CD20  (Rituximab) | - 18F-FDG PET/CT × 1:   Baseline | - Visually (Bone Marrow involvement) | OS & PFS | - Sn & Sp were 94% and 100% for PET-CT and 40% and 100% for marrow biopsy. - BM involvement identified by PET/CT and positive biopsy conferred significantly inferior PFS. |
| [Kim *et al.*](file:///C:\Users\AK\Documents\Meta\ref\Lymphoma\new\Intra-patient%20Variability%20of%20FDG%20Standardized%20Uptake%20Values.pdf)  (2016) *(51)* | NHL: 60  DLBCL | Anti-CD20  (Rituximab) | - 18F-FDG PET/CT × 3:   Baseline  Interim  EOT | - Quantitative ( MTV & TLG of tumor, SUVmax & SUVmean of reference organs) | NA | - The SUV of the liver was not significantly changed during treatment course of the DLBCL patients, whereas the SUV of mediastinal blood pool decreased slightly in interim FDG PET/CT scan. |
| [Kim *et al.*](file:///C:\Users\AK\Documents\Meta\ref\Lymphoma\new\Prognostic%20Value%20of%20Metabolic%20Tumor%20Volume%20Estimated.pdf)  *^a^*  (2014) *(52)* | NHL: 96  DLBCL | Anti-CD20  (Rituximab) | - 18F-FDG PET/CT × 1:   Baseline | - Quantitative (MTV) | OS & PFS | - Baseline MTV measured by FDG-PET/CT is a potential predictor of survival in patients with DLBCL treated with Rituximab. |
| [Kim](file:///C:\Users\AK\Documents\Meta\ref\Lymphoma\Total%20Lesion%20Glycolysis%20in%20Positron%20Emission%20Tomography%20Is%20a.pdf) *et al.* *^a^*  (2013) *(53)* | NHL: 140  Newly diagnosed DLBCL | Anti-CD20  (Rituximab) | - 18F-FDG PET/CT × 1:   Baseline | - Quantitative (TLG & SUVmax) | OS & PFS | - TLG at the threshold of 50% was significantly associated with treatment outcomes, unlike SUVmax. |
| [Kitajima](file:///C:\Users\AK\Documents\Meta\ref\Lymphoma\Predictive%20value%20of%20interim%20FDG-PET-CT%20findings%20in%20patients%20with.pdf) *et al.* *^b, c^*  (2019) *(54)* | NHL: 80  DLBCL | Anti-CD20  (Rituximab) | - 18F-FDG PET/CT × 3:   Baseline  Interim (2-4 cycles)  EOT (after completion) | - DS | PFS | - Interim FDG-PET/CT findings may be useful for determining disease status, though are not recommended for treatment decisions as part of routine clinical practice. - Interim and EOT PET were significantly correlated with PFS. |
| [Kocurek](file:///C:\Users\AK\Documents\Meta\ref\Lymphoma\Primary%20mediastinal%20B%20cell%20lymphoma%20%20%20metabolic%20and%20anatomical%20features%20in%2018FDG%20PET%20CT%20and%20response%20to%20therapy.pdf) *et al.*  (2016) *(55)* | NHL: 7  PMBCL | Anti-CD20  (Rituximab) | - 18F-FDG PET/CT × 3:   Baseline  Interim (3 cycles)  EOT (after completion) | - Quantitative (SUVmax) - DS | OS & PFS | - It seems that the ΔSUVmax was easier in implementation and had a more significant impact than other parameters. |
| [Kong *et al.*](file:///C:\Users\AK\Documents\Meta\ref\Lymphoma\new\Predictive%20Significance%20of%20a%20New%20Prognostic%20Score%20for.pdf)  *^b^*  (2016) *(56)* | NHL: 105  DLBCL | Anti-CD20  (Rituximab) | - 18F-FDG PET/CT × 3:   Baseline  Interim (3 cycles)  EOT (after completion) | - DS | OS & PFS | - In DLBCL patients, interim 18F-FDG PET/CT could provide independent significant prognostic information. |
| [Kostakoglu *et al.*](file:///C:\Users\AK\Documents\Meta\ref\Lymphoma\new\End-of-treatment%20PET%20CT%20predicts%20PFS%20and%20OS%20in%20DLBCL%20after%20first-line%20treatment.pdf)  *^c^*  (2021) *(57)* | NHL: 1118  DLBCL | Anti-CD20  (Rituximab)/Obinutuzumab) | - 18F-FDG PET/CT × 2:   Baseline  EOT (after completion) | - Lugano | OS & PFS | - In the GOYA study complete response in EOT FDG PET is an independent predictor of PFS and OS and could be a promising prognostic factor in DLBCL. |
| [Leppä *et al.*](file:///C:\Users\AK\Documents\Meta\ref\Lymphoma\new\Patients%20with%20high-risk%20DLBCL%20benefit%20from%20dose-dense.pdf)  *^c^*  (2020) *(59)* | NHL: 119  DLBCL | Anti-CD20  (Rituximab) | - 18F-FDG PET/CT × 1:   EOT | - DS | OS & PFS | - The 80% of the patients with a DS of 1-3 on EOT FDG PET scan achieved long-term metabolic remission, and 42% of the patients with DS of 5 relapsed. In contrast, the outcome of those with DS of 4 was comparable to those with negative PET. |
| [Mamot](file:///C:\Users\AK\Documents\Meta\ref\Lymphoma\Final%20Results%20of%20a%20Prospective%20Evaluation%20of%20the%20Predictive.pdf) *et al.* *^b, c^*  (2015) *(60)* | NHL: 138  DLBCL | Anti-CD20  (Rituximab) | - 18F-FDG PET/CT × 3:   Baseline  Interim (2-4 cycles)  EOT | - Quantitative (SUVmax) - DS | OS & PFS | - The interim PET/CT scanning was not ready for clinical use to guide treatment management in individual patients. - PFS was significantly shorter for interim and EOT PET-positive compared with PET-negative patients, however OS was not significantly different. |
| [Mayerhoefer](file:///C:\Users\AK\Documents\Meta\ref\Lymphoma\Pre-Therapeutic%20Total%20Lesion%20Glycolysis%20on%20%5b18F%5dFDG-PET%20Enables%20Prognostication%20of%202%20Year%20Progression%20Free%20Survival%20in%20MALT%20Lymphoma%20Patients%20Treated%20with%20CD20%20Antibody%20Based%20Immunotherapy.pdf) *et al.* *^a^*  (2019) *(61)* | NHL: 35  Newly diagnosed MALT lymphoma | Anti-CD20  (Rituximab) | - 18F-FDG PET/CT × 1:   Baseline | - Quantitative (MTV, TLG, SUVmax & SUVmean) | PFS | - In univariate analysis, TLG and MTV had significant association with PFS. |
| [Mayerhoefer](file:///C:\Users\AK\Documents\Meta\ref\Lymphoma\Radiomic%20features%20of%20glucose%20metabolism%20enable%20prediction.pdf) *et al.*  (2019) *(62)* | NHL: 107  Newly diagnosed MCL | Anti-CD20  (Rituximab) | - FDG-PET/CT × 1:   Baseline | - Quantitative (MTV, TLG, SUVmax & SUVmean) | PFS | - SUVmean and Entropy had significant correlation with PFS. - The best results may be achieved using a combination of metabolic, clinical and laboratory parameters. |
| [Mayerhoefer](file:///C:\Users\AK\Documents\Meta\ref\Lymphoma\Ultra-early%20response%20assessment%20in%20lymphoma%20treatment%2018F%20FDG%20PET%20MR%20captures%20changes%20in%20glucose%20metabolism%20and%20cell%20density%20within%20the%20first%2072%20hours%20of%20treatment.pdf) *et al.*  (2018) *(63)* | HL: 11  NHL: 47 | Anti-CD20  (Rituximab) | - 18F-FDG PET/MRI × 3:   Baseline  Early 1 (48-72 h)  Early 2 (1 w) | - Quantitative (MTV, SUVmax & SUVmean) | - | - In lymphoma patients, FDG PET/MR can reveal treatment-induced changes in glucose consumption and cell density as early as 48-72 h after initiation of treatment. |
| [Mayerhoefer](file:///C:\Users\AK\Documents\Meta\ref\Lymphoma\Can%20Interim%2018F-FDG%20PET%20or%20Diffusion-Weighted%20MRI%20Predict%20End-of-Treatment%20Outcome%20in%20FDG-Avid%20MALT%20Lymphoma%20After%20Rituximab-Based%20Therapy.pdf) *et al.*  (2016) *(64)* | NHL: 15  MALT lymphoma | Anti-CD20  (Rituximab) | - 18F-FDG PET/CT & MRI × 3:   Baseline  Interim (3 cycles)  EOT (6 cycles) | - Quantitative (SUVmax & SUVmean) | Histology & CE-CT | - Both quantitative interim 18F-FDG PET and DWI may be useful to predict complete remission at EOT in MALT lymphoma. |
| [Melani](file:///C:\Users\AK\Documents\Meta\ref\Lymphoma\End-of-treatment%20and%20serial%20PET%20imaging%20in%20primary%20mediastinal%20B-cell%20lymphoma%20following%20dose-adjusted%20EPOCH-R%20a%20paradigm%20shift%20in%20clinical%20decision%20making.pdf) *et al.* *^c^*  (2018) *(65)* | NHL: 93  PMLBCL | Anti-CD20  (Rituximab) | - FDG-PET/CT × 1:   EOT (3 w after completion) | - Quantitative (MTV, TLG & SUVmax) - DS | OS & PFS | - Patients with a negative EOT FDG-PET had a significantly better PFS and OS compared to patients with positive scans. |
| [Micallef *et al.*](file:///C:\Users\AK\Documents\Meta\ref\Lymphoma\new\Epratuzumab%20with%20rituximab,%20cyclophosphamide,%20doxorubicin,%20vincristine,%20and.pdf)  (2011) *(66)* | NHL: 76  DLBCL | Anti-CD20  (Rituximab)  Anti-CD22 | - FDG-PET/CT × 2:   Interim (2 cycles)  EOT (6 cycles) | - Visually | OS & PFS | - Interim PET negativity was not associated with a statistically significant improvement in PFS or OS; However, EOT PET negativity was associated with a statistically significant improvement in PFS & OS. |
| [Mikhaeel](file:///C:\Users\AK\Documents\Meta\ref\Lymphoma\Combination%20of%20baseline%20metabolic%20tumour%20volume%20and%20early.pdf) *et al.* *^a, b^*  (2016) *(67)* | NHL: 147  DLBCL | Anti-CD20  (Rituximab) | - FDG-PET/CT × 2:   Baseline  Interim (2 cycles) | - Quantitative (MTV, TLG & SUVmax) - DS | OS & PFS | - The baseline MTV and TLG parameters significantly correlated with PFS. - Combining baseline MTV parameter and interim FDG PET data improved the predictive power of interim scan and could define poor-prognosis patients. |
| [Minamimoto *et al.*](file:///C:\Users\AK\Documents\Meta\ref\Lymphoma\new\Diffuse%20Large%20B-Cell%20Lymphoma.pdf)  (2016) *(68)* | NHL: 46  DLBCL | Anti-CD20  (Rituximab) | - 18F-FDG PET/CT × 3:   Baseline  Interim  EOT   - 18F-FLT PET/CT × 2:   Interim  EOT | - Quantitative (MTV, TLG & SUVmax) - IHP - EORTC - PERCIST - DS | Histological evaluation | - Interim FLT PET/CT had a significantly higher PPV than FDG PET/CT for treatment response assessment in DLBCL. |
| [Mir](file:///C:\Users\AK\Documents\Meta\ref\Lymphoma\Baseline%20SUVmax%20did%20not%20predict%20histological%20transformation%20in%20follicular%20lymphoma%20in%20the%20phase%203%20GALLIUM%20study.pdf) *et al.*  (2020) *(69)* | NHL: 549  FL | Anti-CD20  (Rituximab/Obinutuzumab) | - FDG-PET/CT × 2:   Baseline  EOT | - Quantitative (SUVmax, SUVrange) - IHP | Histological transformation | - In the GALLIUM study neither baseline SUVmax nor baseline SUVrange predicted histological transformation. |
| [Morschhauser *et al.*](file:///C:\Users\AK\Documents\Meta\ref\Lymphoma\new\A%20phase%20II%20study%20of%20venetoclax%20plus%20R-CHOP%20as%20first-line%20treatment%20for%20patients%20with%20diffuse%20large.pdf)  (2021) *(71)* | NHL: DLBCL | Anti-CD20  (Rituximab) | - 18F-FDG PET/CT × 1:   EOT | - Lugano | OS & PFS | - Only the Lugano response categories are reported. |
| [Moskowitz *et al.*](file:///C:\Users\AK\Documents\Meta\ref\Lymphoma\new\Risk-Adapted%20Dose-Dense%20Immunochemotherapy.pdf)  (2010) *(72)* | NHL: 98  DLBCL | Anti-CD20  (Rituximab) | - FDG-PET/CT × 3:   Baseline  Interim  EOT | - Visually | OS & PFS | - Neither interim nor EOT FDG-PET evaluation predicted outcome. |
| [OÑATE‑OCAÑA *et al.*](file:///C:\Users\AK\Documents\Meta\ref\Lymphoma\new\Metabolic%20tumor%20volume%20changes%20assessed%20by%20interval.pdf)  *(73)* | NHL: 51  DLBCL | Anti-CD20  (Rituximab) | - 18F-FDG PET/CT × 3:   Baseline  Interim (3 cycles)  EOT | - Quantitative (SUVmax & MTV) - DS | OS & PFS | - Assessment of quantitative parameters from interim FDG PET/CT scans combined with clinical variables can predict complete response at the end of treatment. |
| [Pinnix *et al.*](file:///C:\Users\AK\Documents\Meta\ref\Lymphoma\new\Positron%20emission%20tomography–computed%20tomography%20predictors%20of.pdf) *^a, c^*  (2018) *(74)* | NHL: 65  PMBCL | Anti-CD20  (Rituximab) | - 18F-FDG PET/CT × 2:   Baseline  EOT | - Quantitative (SUVmax, MTV & TLG) - DS | PFS | - In univariate analysis, TLG and MTV had significant association with PFS. - Combining baseline TLG & EOT DS identified patients at increased risk of progression. |
| [Rutherford](file:///C:\Users\AK\Documents\Meta\ref\Lymphoma\Impact%20of%20bone%20marrow%20biopsy%20on%20response%20assessment%20in.pdf) *et al.*  (2020) *(75)* | NHL: 402 | Anti-CD20  (Rituximab /Obin) | - 18F-FDG PET/CT × 1:   EOT | - Lugano | PFS | - In GALLIUM and GOYA studies, respectively, 4.7% of FL patients and 7.1% of DLBCL patients had a repeat BMB result that altered response assessment results with FDG PET/CT. These findings indicated that bone marrow evaluation appears to add value to FDG PET/CT response assessment in DLBCL. |
| [Sasanelli](file:///C:\Users\AK\Documents\Meta\ref\Lymphoma\Pretherapy%20metabolic%20tumour%20volume%20is%20an%20independent%20predictor.pdf) *et al.* *^a^*  (2014) *(76)* | NHL: 114  Newly diagnosed DLBCL | Anti-CD20  (Rituximab) | - 18F-FDG PET/CT × 1:   Baseline | - Quantitative (MTV, TLG & SUVmax) | OS & PFS | - Pretherapy MTV is an independent predictor of PFS and OS in patients with DLBCL. |
| [Schmitz](file:///C:\Users\AK\Documents\Meta\ref\Lymphoma\Dynamic%20risk%20assessment%20based%20on%20positron%20emissiontomography%20scanning%20in%20diffuse%20large%20B-cell%20lymphomaPost-hoc%20analysis%20from%20the%20PETAL%20trial.pdf) *et al.* *^a, b^*  (2020) *(77)* | NHL: 510  AL | Anti-CD20  (Rituximab) | - 18F-FDG PET/CT × 2:   Baseline  Interim (2 cycles) | - Quantitative (MTV & SUVmax) | OS & PFS | - In the PETAL trial both baseline TMTV and interim PET response predicted outcome. |
| [Senjo *et al.*](file:///C:\Users\AK\Documents\Meta\ref\Lymphoma\new\High%20metabolic%20heterogeneity%20on%20baseline%2018FDG-PET%20CT%20scan%20as%20a%20poor.pdf)  *^a^*  (2020) *(78)* | NHL: 150  DLBCL | Anti-CD20  (Rituximab) | - 18F-FDG PET/CT × 1:   Baseline | - Quantitative (MTV & metabolic heterogenity) | OS & PFS | - Metabolic heterogenity on baseline 18FDG-PET/CT scan predicts treatment outcomes for patients with newly diagnosed DLBCL. - Baseline metabolic heterogenity in combination with baseline MTV provided a new approach to the risk stratification of patients with newly diagnosed DLBCL. |
| [Song *et al.*](file:///C:\Users\AK\Documents\Meta\ref\Lymphoma\new\Prognostic%20signifcance%20of%20interim.pdf) *^b^*  (2020) *(80)* | NHL: 146  MZL | Anti-CD20  (Rituximab) | - 18F-FDG PET/CT × 1:   Baseline  Interim | - Quantitative (ΔSUVmax) - DS | PFS | - The interim PET/CT response assessment based on the DS is useful factor for predicting PFS of MZL patients. |
| [Song](file:///C:\Users\AK\Documents\Meta\ref\Lymphoma\High%20total%20metabolic%20tumor%20volume%20in%20PET%20CT%20predicts%20worse.pdf) *et al.* *^a^*  (2016) *(81)* | NHL: 107  denovo DLBCL | Anti-CD20  (Rituximab) | - 18F-FDG PET/CT × 2:   Baseline  EOT | - Quantitative (MTV & Intramedullary MTV) | OS & PFS | - Baseline MTV had significant correlation with PFS and OS. |
| [Song *et al*](file:///C:\Users\AK\Documents\Meta\ref\Lymphoma\new\Prediction%20of%20Central%20Nervous%20System%20Relapse%20of%20Diffuse.pdf)*.*  (2015) *(82)* | NHL: 180  DLBCL | Anti-CD20  (Rituximab) | - 18F-FDG PET/CT × 1:   Baseline | - Quantitative (MTV & TLG) | PFS (for CNS relapse) | - High TLG on FDG PET/CT is the most significant predictor of CNS relapse in untreated DLBCL patients. |
| [Song](file:///C:\Users\AK\Documents\Meta\ref\Lymphoma\Clinical%20significance%20of%20metabolic%20tumor%20volume%20by%20PETCTin%20stages%20II%20and%20III%20of%20diffuse%20large%20B%20cell%20lymphomawithout%20extranodal%20site%20involvement.pdf) *et al.* *^a^*  (2012) *(83)* | NHL: 169  denovo nodal DLBCL | Anti-CD20  (Rituximab) | - 18F-FDG PET/CT × 1:   Baseline | - Quantitative (MTV) - rIWC | OS & PFS | - Baseline MTV had significant correlation with PFS and OS. - Assessment of MTV by PET had more potential predictive power than Ann Arbor stage in the patients that received R-CHOP. |
| [Song](file:///C:\Users\AK\Documents\Meta\ref\Lymphoma\Prognostic%20value%20of%20metabolic%20tumor%20volume%20on.pdf) *et al.* *^a^*  (2012) *(84)* | NHL: 165  Extranodal GI DLBCL | Anti-CD20  (Rituximab) | - 18F-FDG PET/CT × 2:   Baseline  EOT (3 w after completion) | - Quantitative (MTV & SUVmax) - rIWC | OS & PFS | - Baseline MTV and SUVmax had significant correlation with PFS and OS. - MTV was a better predictor for survival compared to SUVmax. |
| [Sun](file:///C:\Users\AK\Documents\Meta\ref\Lymphoma\Risk%20Stratification%20Of%20Diffuse%20Large%20B-Cell%20Lymphoma%20With%20Interim%20PET%20CT%20By%20Combining%20Deauville%20Scores%20And%20International%20Prognostic%20Index.pdf) *et al.* *^b, c^*  (2019) *(85)* | NHL: 111  DLBCL | Anti-CD20  (Rituximab) | - 18F-FDG PET/CT × 1:   Interim (3/4 cycles)  or  EOT (5/6 courses) | - DS | PFS | - The IPI adds strength to DS in interim PET to detect patients with good or poor prognosis. |
| [Swinnen *et al*](file:///C:\Users\AK\Documents\Meta\ref\Lymphoma\new\Response-Adapted%20Therapy%20for%20Aggressive%20non-Hodgkin's.pdf)*.*  (2015) *(86)* | NHL: 74  Stage III or IV or II bulky DLBCL | Anti-CD20  (Rituximab) | - 18F-FDG PET/CT × 3:   Baseline  Interim (3 cycles) | - Visually (DS?) | OS & PFS | - Treatment modification based on interim FDG PET/CT imaging should remain confined to clinical trials. |
| [Takasaki *et al.*](file:///C:\Users\AK\Documents\Meta\ref\Lymphoma\new\Post-treatment%20PET–CT%20Findings%20may%20Predict%20the%20Prognosis.pdf)  (2015) *(87)* | NHL: 26  DLBCL with bulky mass | Anti-CD20  (Rituximab) | - 18F-FDG PET/CT × 2:   Baseline  EOT | - Visually | OS & PFS | - EOT FDG PET/CT findings may have predictive value in DLBCL patients with a bulky mass. |
| [Tateishi](C:\\Users\\AK\\Documents\\Meta\\ref\\Lymphoma\\new\\Prognostic significance of metabolic tumor burden.pdf) *[et al.](C:\\Users\\AK\\Documents\\Meta\\ref\\Lymphoma\\new\\Prognostic significance of metabolic tumor burden.pdf)*  (2015) *(88)* | NHL: 55  DLBCL | Anti-CD20  (Rituximab) | - 18F-FDG PET/CT × 3:   Baseline  Interim (2 cycles)  EOT (after completion) | - Lugano - Quantitative (MTV, TLG & SUVmax) | PFS | - The percent change TLG can be used to quantify the response to treatment and can predict PFS after the last treatment. |
| [Toledano](file:///C:\Users\AK\Documents\Meta\ref\Lymphoma\Comparison%20of%20therapeutic%20evaluation%20criteria.pdf) *et al.* *^b, c^*  (2019) *(89)* | NHL: 181  DLBCL | Anti-CD20  (Rituximab) | - 18F-FDG PET/CT × 3:   Baseline  Interim  EOT (after completion) | - Quantitative (SUVmax & Tumor/Liver ratio) - DS | OS & PFS | - A tumor to liver ratio of 1.4 could be a robust prognostic factor in DLBCL patients on interim PET and EOT PET. |
| [Trotman](file:///C:\Users\AK\Documents\Meta\ref\Lymphoma\Prognostic%20value%20of%20end%20of%20induction%20PET%20response%20after.pdf) *et al.* *^c^*  (2018) *(90)* | NHL: 595  Anti-CD20 positive lymphoma | Anti-CD20  (Rituximab /Obinutuzumab) | - 18F-FDG PET/CT × 2:   Baseline  EOT (after completion) | - IHP - Lugano | OS & PFS | - Results suggest that PET is a better imaging modality than CE-CT for response assessment after first-line immunochemotherapy in patients with follicular lymphoma. - FDG PET/CT assessment according to the Lugano criteria provided a platform for response-adapted therapeutic management. |
| [Vaxman](file:///C:\Users\AK\Documents\Meta\ref\Lymphoma\FDG%20PET%20CT%20as%20a%20diagnostic%20and%20prognostic%20tool%20for%20the%20evaluation%20of%20marginal%20zone%20lymphoma.pdf) *et al.* *^c^*  (2019) *(91)* | NHL: 110  Untreated MZL | Anti-CD20  (Rituximab) | - 18F-FDG PET/CT × 3:   Baseline  Interim (3 cycles)  EOT (6 cycles) | - Quantitative (SUVmax) - DS | OS & PFS | - EOT PET/CT was found to be a significant predictor for PFS. |
| [Vercellino *et al.*](file:///C:\Users\AK\Documents\Meta\ref\Lymphoma\new\High%20total%20metabolic%20tumor%20volume%20at%20baseline%20predicts.pdf)  *^a^*  (2020) *(92)* | NHL: 301  DLBCL | Anti-CD20  (Rituximab) | - 18F-FDG PET/CT × 1:   Baseline | - Quantitative (MTV) | OS & PFS | - High TMTV at baseline was significantly associated with inferior PFS and OS in DLBCL patients. |
| [Wang *et al.*](file:///C:\Users\AK\Documents\Meta\ref\Lymphoma\new\Prognostic%20value%20of%20interim%20fluorodeoxyglucose.pdf) *^b^*  (2018) *(96)* | NHL: 44  DLBCL | Anti-CD20  (Rituximab) | - 18F-FDG PET/CT × 2:   Baseline  Interim   - 18F-FLT PET/CT × 2:   Baseline  Interim | - Quantitative (ΔSUVmax) - DS | OS & PFS | - Interim FLT PET/CT had higher accuracy and specificity compared to standard FDG PET/CT scan. |
| [Wei](file:///C:\Users\AK\Documents\Meta\ref\Lymphoma\A%20single%20center%20experience%20rituximab%20plus%20cladribine%20is%20an.pdf) *et al.*  (2017) *(97)* | NHL: 8  BALT | Anti-CD20  (Rituximab) | - 18F-FDG PET/CT× 3:   Baseline  Interim  EOT | - Quantitative (SUVmax) - IWGRRC | OS & PFS | - Rituximab and cladribine therapy demonstrated high activity in chemotherapy-naïve patients with advanced stages of bronchial-associated lymphoid tissue lymphoma. |
| [Wong-Sefidan](file:///C:\Users\new\18F%5d%20Positron%20emission%20tomography%20response%20after%20rituximab.pdf) *et al.*  (2017) *(98)* | NHL: 447  FL | Anti-CD20  (Rituximab) | - 18F-FDG PET/CT× 2:   Baseline  EOT (12 w) | - Visually | OS & PFS | - FDG PET/CT provided important prognostic information after treatment initiation. - Positive EOT PET/CT imaging was correlated with inferior OS. |
| [Yim *et*](file:///C:\Users\Early%20risk%20stratification%20for%20diffuse%20large%20B-cell%20lymphoma%20integrating.pdf) *al.* *^b^*  (2019) *(99)* | NHL: 220  Newly diagnosed DLBCL | Anti-CD20  (Rituximab) | - 18F-FDG PET/CT × 3:   Baseline  Interim (3 cycles)  EOT | - DS | OS & PFS | - Early risk stratification using interim DS predicted the risk of PFS and OS in patients with DLBCL. |
| [Younes *et al.*](file:///C:\Users\AK\Documents\Meta\ref\Lymphoma\new\Phase%202%20study%20of%20rituximab%20plus%20ABVD%20in%20patients%20with%20newly%20diagnosed.pdf)  *^b^*  (2012) *(100)* | HL: 65 | Anti-CD20  (Rituximab) | - 18F-FDG PET/CT × 3:   Baseline  Interim (3 cycles)  EOT | - Visually | OS & PFS | - The 5 year PFS in patients with negative interim PET scans and those with positive interim PET scans were 91% and 77%, respectively. |
| [Zhang *et al.*](file:///C:\Users\AK\Documents\Meta\ref\Lymphoma\new\A%20better%20prediction%20of%20progression‐free%20survival%20in%20diffuse%20large.pdf)  *^a^*  (2019) *(101)* | NHL: 220  Newly diagnosed DLBCL | Anti-CD20  (Rituximab) | - 18F-FDG PET/CT × 2:   Baseline  Interim (2/4 cycles) | - Quantitative (MTV, TLG, SUVmax & SUVmean) - Lugano | PFS | - Baseline MTV, TLG and SUVmax had significant correlation with PFS. - Combining the ΔSUVmax and baseline TLG showed the best screening ability among other baseline and interim PET/CT parameters. |
| [Zhang *et al.*](file:///C:\Users\AK\Documents\Meta\ref\Lymphoma\new\Use%20of%20subsequent%20PETCT%20in%20diffuse%20large%20B-cell%20lymphoma%20patients%20in%20complete%20remission%20following%20primary%20therapy.pdf) *^b^*  (2015) *(102)* | NHL: 197  DLBCL | Anti-CD20  (Rituximab) | - 18F-FDG PET/CT × 2:   Interim (2&4 cycles)  EOT | - IHP | OS & PFS | - Interim PET/CT should be performed after 2 rather than 4 cycles of immunochemotherapy in DLBCL patient |
| [Zhao *et al.*](file:///C:\Users\AK\Documents\Meta\ref\Lymphoma\new\Prognostic%20value%20of%20the%20baseline%2018F‑FDG%20PETCT%20metabolic%20tumour.pdf) *^a^*  (2021) *(103)* | NHL: 87  DLBCL | Anti-CD20  (Rituximab) | - 18F-FDG PET/CT × 1:   Baseline | - Quantitative (MTV, TLG, SUVmax & SUVmean) | OS & PFS | - Baseline MTV had significant correlation with PFS and OS. |
| [Zhou](file:///C:\Users\AK\Documents\Meta\ref\Lymphoma\Prognostic%20values%20of%20baseline,%20interim%20and%20end-of%20therapy%2018F-FDG%20PET%20CT%20in%20patients%20with%20follicular%20lymphoma.pdf) *et al.* *^a, b, c^*  (2019) *(104)* | NHL: 84  FL | Anti-CD20  (Rituximab) | - 18F-FDG PET/CT × 3:   Baseline  Interim (2-4 cycles)  EOT | - Quantitative (MTV, TLG & SUVmax) - IHP - DS | OS & PFS | - Baseline MTV, TLG and SUVmax had significant correlation with PFS. - Baseline MTV and TLG had significant correlation with OS. |
| [Zhou](file:///C:\Users\AK\Documents\Meta\ref\Lymphoma\Prognostic%20value%20of%20total%20lesion%20glycolysis%20of%20baseline.pdf) *et al.* *^a^*  (2016) *(105)* | NHL: 91  Newly diagnosed DLBCL | Anti-CD20  (Rituximab) | - 18F-FDG PET/CT × 1:   Baseline | - Quantitative (MTV, TLG, SUVmax & Liver SUVmean) | OS & PFS | - Baseline MTV and TLG had significant correlation with PFS and OS. |
| [Zinzani](file:///C:\Users\Midtreatment18F-FluorodeoxyglucosePositron-Emission%20Tomography%20inAggressive%20Non-Hodgkin%20Lymphoma.pdf) *et al.*  (2011) *(106)* | NHL: 91  Newly diagnosed PMLBCL & DLBCL | Anti-CD20  (Rituximab) | - 18F-FDG PET/CT × 3:   Baseline  Interim (4-6 w or 3 cycles)  EOT | - Visually | OS & PFS | - The interim PET may have a role in helping physicians for decision making. |

*^a.^ Participate in baseline metabolic parameters meta-analysis.*

*^b.^ Participate in interim response assessment meta-analysis.*

*^c.^ Participate in EOT response assessment meta-analysis.*

HL, Hodgkin lymphoma; NHL, Non-Hodgkin lymphoma; DLBCL, Diffuse large B cell lymphoma; PMLBCL , Primary mediastinal large B cell lymphoma; HGBCL, High grade B cell lymphoma FL, follicular lymphoma; MALT, mucosa-associated lymphoid tissue lymphoma; MCL; Mantle cell lymphoma; MZL, Marginal zone lymphoma; BALT lymphoma, Bronchial-associated lymphoid tissue lymphoma; CTL therapy, Cytotoxic T Lymphocyte Therapy; DC, dendritic cell; EOT, end of treatment; w, week; mo, month; DS, Deauville Score; IHP, international Harmonization Project; rIWC, revised International Workshop Criteria; MTV, metabolic tumor volume; TLG, total lesion glycolysis; BOR, best overall response; CE-CT, contrast enhanced CT; OS, overall survival; PFS, progression free survival.
