# Supplementary material for: The value of FDG PET/CT imaging in outcome prediction and response assessment of lymphoma patients treated with immunotherapy: a meta-analysis and systematic review
Source: Eur J Nucl Med Mol Imaging. 2022 Aug 6;49(13):4661–76. doi: 10.1007/s00259-022-05918-2 (PMC9606078; doi:10.1007/s00259-022-05918-2)
Supplement: Supplementary file 3 — Supplementary file3 (DOCX 22 KB) [file 259_2022_5918_MOESM3_ESM.docx]

Table 3. Basic characteristic of 8 included articles in lymphoma patients treated with immune checkpoint inhibitors.

| Author  (year) | Lymphoma type, sample size | Treatment | Imaging intervals | Response assessment criteria | Clinical outcome | Conclusion |
| --- | --- | --- | --- | --- | --- | --- |
| [Allen](file:///C:\Users\AK\Documents\Meta\ref\Lymphoma\Pembrolizumab%20followed%20by%20AVD%20in%20untreated%20early%20unfavorable%20and%20advanced%20stage.pdf) *et al.*  (2020) *(17)* | HL: 30 | Pembrolizumab + AVD | - 18F-FDG PET/CT × 4:   Baseline  Interim (3 cycles of PEM)  Late (2 cylces of AVD)  EOT | - Quantitave (MTV) - Lugano - DS | OS & PFS | - Baseline MTV was not associated with achieving a CMR after pembrolizumab monotherapy or greater than 90% reduction in MTV in early response assessment. |
| [Bartlett *et al.*](file:///C:\Users\AK\Documents\Meta\ref\Lymphoma\new\A%20phase%201b%20study%20of%20AFM13%20in%20combination%20with%20pembrolizumab%20in%20patients%20with%20relapsed.pdf)  (2020)  *(19)* | HL: 30 | Pembrolizumab | - FDG-PET/CT × 2:   Baseline  FU every 12 W | - Lugano | BOR | - 84% of patient was responder (CMR + PMR) to treatment and reached their best response at 13 to 26 weeks after therapy initiation and their response duration was between 9 [95% CI: 8.4, not estimable] for CMR to 10.4 for PMR [95% CI: 2.8, 10.4] months. |
| [Castello](C:\\Users\\AK\\Documents\\Meta\\ref\\Lymphoma\\18F-FDG PET CT for response assessment in Hodgkin lymphoma undergoing immunotherapy with checkpoint inhibitors.pdf) *[et al.](C:\\Users\\AK\\Documents\\Meta\\ref\\Lymphoma\\18F-FDG PET CT for response assessment in Hodgkin lymphoma undergoing immunotherapy with checkpoint inhibitors.pdf)*  (2019) *(24)* | HL: 43 | Pembrolizumab/ Nivolumab | - 18F-FDG PET/CT × 3:   Baseline  Early (8-w)  Interim (17-w) | - Quantitative (MTV, TLG, SUVmax & SUVmean) - DS - Lugano - LYRIC | OS, PFS & BOR | - In early evaluation, the reduction in tumor burden was statistically higher in responders. - Visual analysis by DS showed a significant difference within the responder group compared to the other. - Significant reduction in both tumor metabolism (ΔSUVmax) and tumor burden (ΔMTV and ΔTLG) between responders and nonresponders. |
| [Chen](C:\\Users\\AK\\Documents\\Meta\\ref\\Lymphoma\\Early 18F FDG PET CT response predicts survival in Relapsed Refractory Hodgkin Lymphoma treated with Nivolumab.pdf) *[et al.](C:\\Users\\AK\\Documents\\Meta\\ref\\Lymphoma\\Early 18F FDG PET CT response predicts survival in Relapsed Refractory Hodgkin Lymphoma treated with Nivolumab.pdf)*  (2019)*(28)* | HL: 45 | Nivolumab | - 18F-FDG PET/CT × 2:   Baseline  Early (3-mo) | - Lugano - LYRIC | OS, PFS & BOR | - According to the early response assessment with FDG PET both Lugano and LYRIC criteria classified patients as CMR, PMR, PMD and NMR, similarly. - The 2 year OS probability was significantly different in patients with PMD, NMR or PMR, and CMR. |
| [Dercle](C:\\Users\\AK\\Documents\\Meta\\ref\\Lymphoma\\18F-FDG PET and CT Scans Detect New Imaging Patterns of.pdf) *[et al.](C:\\Users\\AK\\Documents\\Meta\\ref\\Lymphoma\\18F-FDG PET and CT Scans Detect New Imaging Patterns of.pdf)*  (2018) *(36)* | HL: 16 | Pembrolizumab/Nivolumab | - 18F-FDG PET/CT &   CE-CT:  Baseline  FU (every 3-mo) | - Quantitative (MTV, TLG, SUVmax, SUVmean & SUVmax of healthy lymphoid tissue) - DS - Lugano - LYRIC | BOR  (IHPC & LYRIC) | - The most accurate biomarker of early imaging for prediction of BOR was MTV. - The 5 point DS was not significantly different between refractory and responding patients, whereas responders had significantly greater decrease in SUVmean and SUVmax. - Significant increase in spleen glucose consumption (ΔSUVmax) was observed in responders. |
| [Lepik *et al.*](file:///C:\Users\AK\Documents\Meta\ref\Lymphoma\new\A%20Phase%202%20Study%20of%20Nivolumab%20Using%20a%20Fixed%20Dose.pdf)  (2020) *(58)* | HL: 30 | Nivolumab | - FDG-PET/CT × 2:   Baseline  FU (every 3-mo) | - LYRIC - DS | OS, PFS & BOR | - According to the LYRIC criteria patients with indeterminate response have outcomes comparable to patients with partial response and stable disease. - Achievement of negative PET complete response at 3 months after the treatment initiation significantly affected PFS. |
| [Mokrane](C:\\Users\\AK\\Documents\\Meta\\ref\\Lymphoma\\Performance of CT Compared with 18F-FDG PET in.pdf) *[et al.](C:\\Users\\AK\\Documents\\Meta\\ref\\Lymphoma\\Performance of CT Compared with 18F-FDG PET in.pdf)*  (2020) *(70)* | HL: 45 | Nivolumab | - 18F-FDG PET/CT × 2:   Baseline  Early | - LYRIC criteria - Lugano | OS & PFS | - The early response assessment using FDG PET/CT was associated with OS and PFS. |
| [Voltin](file:///C:\Users\new\18F-FDG%20PET%20CT%20for%20Early%20Response%20Assessment%20in%20Diffuse.pdf) *et al.*  (2019) *(94)* | HL: 59 | Nivolumab | - 18F-FDG PET/CT × 2:   Baseline  Interim | - Quantitative (MTV) - DS | BOR | - MTV may have the potential to accurately measure response to ICI. |

HL, Hodgkin lymphoma; NHL, Non-Hodgkin lymphoma; CAM, camrelizumab; SIN, sintilimab; PID, Pidilizumab; BV, Brentuximab; EOT, end of treatment; w, week; mo, month; DS, Deauville Score; RRCML, revised response criteria for malignant lymphoma; IHP, international Harmonization Project; rIWC, revised International Workshop Criteria; LYRIC, lymphoma Response to Immunomodulatory therapy Criteria; MTV, metabolic tumor volume; TLG, total lesion glycolysis; BOR, best overall response; CE-CT, contrast enhanced CT; OS, overall survival; PFS, progression free survival.
