# Supplementary material for: The value of FDG PET/CT imaging in outcome prediction and response assessment of lymphoma patients treated with immunotherapy: a meta-analysis and systematic review
Source: Eur J Nucl Med Mol Imaging. 2022 Aug 6;49(13):4661–76. doi: 10.1007/s00259-022-05918-2 (PMC9606078; doi:10.1007/s00259-022-05918-2)
Supplement: Supplementary file 4 — Supplementary file4 (DOCX 21 KB) [file 259_2022_5918_MOESM4_ESM.docx]

Table 4. Basic characteristics of the 6 included articles in lymphoma patients treated with cell immunotherapy.

| Authors  (year) | Lymphoma type, sample size | Treatment | Imaging modality & intervals | Response assessment method | Clinical outcome | Conclusion |
| --- | --- | --- | --- | --- | --- | --- |
| [Baudard](file:///C:\Users\AK\Documents\Meta\ref\Lymphoma\Importance%20of%20%5b18F%5dfluorodeoxyglucose-positron%20emission%20tomography%20scanning%20for%20the%20monitoring%20of%20responses%20to%20immunotherapy%20in%20follicular%20lymphoma.pdf) *et al.*  (2007) *(20)* | NHL: 6  FL | DC | - 18F-FDG PET/CT × 2:   Baseline  Early (1 mo after last dose) | - Quantitative (SUVmax) | CT scan | - FDG PET was more sensitive than CT for initial staging, revealing lymph nodes positive on PET but of less than 1 cm of small diameter on CTs. - FDG PET evaluated disease progression with more accuracy than CT. |
| [Derlin *et al.*](file:///C:\Users\AK\Documents\Meta\ref\Lymphoma\new\18F‑FDG%20PETCT%20of%20of‑target%20lymphoid%20organs%20in%20CD19‑targeting.pdf)  (2021) *(37)* | NHL: 10  DLBCL | CAR T-cell | - 18F-FDG PET/CT × 3:   Baseline  Early (1 mo)  Late (3 mo) | - Quantitative (MTV, TLG, SUVmax & SUVmean of tumor and lymphoid organs) - Lugano | PET response at 3 mo | - Baseline metabolic parameters of lymphoma were not significantly correlated with patient outcome. - High baseline glucose metabolism of lymphoma lesions was associated with toxicity. - Early suppression of glucose metabolism in lymphoid organs was associated with poor outcome. |
| [Hart](file:///C:\Users\Use%20of18F-FDG%20positron%20emission%20tomography%20followingallogeneic%20transplantation%20to%20guide%20adoptive%20immunotherapywith%20donor%20lymphocyte%20infusions.pdf) *et al.*  (2005) *(47)* | NHL: 55 | Donor lymphocyte | - 18F-FDG PET/CT × 2:   After bone marrow transplantation  F-U | - NA | NA | - FDG PET/CT could have a role in guiding donor lymphocyte administration and monitoring the immunotherapeutic effect in patients after allogeneic transplantation. |
| [Shah](file:///C:\Users\Early%20positron%20emission%20tomography%20computed%20tomography%20as%20a%20predictor%20of%20response%20after%20CTL019%20chimeric%20antigen%20receptor%20–T-cell%20therapy%20in%20B-cell%20non-Hodgkin%20lymphomas.pdf) *et al.*  (2018) *(79)* | NHL: 7 | CTL therapy | - 18F-FDG PET/CT × 3:   Baseline  Early (1 mo)  EOT (after completion) | - Quantitative (MTV) - DS | DS of EOT PET/CT | - This study suggests that early FDG PET/CT and measurement of total MTV may be effective tools for early response assessment after T cell therapy in patients with NHL. |
| [Vercellino *et al.*](file:///C:\Users\AK\Documents\Meta\ref\Lymphoma\new\Predictive%20factors%20of%20early%20progression%20after%20CAR%20T-cell%20therapy%20in.pdf)  (2020) *(93)* | NHL: 116  DLBCL | Anti-CD19 CAR-T cells | - 18F-FDG PET/CT × 1:   Baseline | - Quantitative (MTV) | OS & PFS | - Univariate analyses for PFS and OS identified high TMTV as risk factors. |
| [Wang *et al.*](file:///C:\Users\AK\Documents\Meta\ref\Lymphoma\Role%20of%20Fluorodeoxyglucose%20Positron%20Emission%20Tomography%20Computed.pdf) *^a^*  (2019) *(95)* | NHL: 19 | Anti-CD19 CAR-T cells | - 18F-FDG PET/CT × 4:   Baseline  FU a 1, 2 & 3 mo | - Quantitative (MTV & TLG) - PERCIST | OS | - The patients with higher baseline disease burden have more severe cytokine release syndrome. - The CAR-T cell therapy is associated with lymphoma pseudoprogression and local immune activation. - Neither baseline MTV nor baseline TLG was significantly associated with OS. |

HL, Hodgkin lymphoma; NHL, Non-Hodgkin lymphoma; CAM, camrelizumab; SIN, sintilimab; PID, Pidilizumab; BV, Brentuximab; EOT, end of treatment; w, week; mo, month; DS, Deauville Score; RRCML, revised response criteria for malignant lymphoma; IHP, international Harmonization Project; rIWC, revised International Workshop Criteria; LYRIC, lymphoma Response to Immunomodulatory therapy Criteria; MTV, metabolic tumor volume; TLG, total lesion glycolysis; BOR, best overall response; CE-CT, contrast enhanced CT; OS, overall survival; PFS, progression free survival.
