# Supplementary material for: The value of FDG PET/CT imaging in outcome prediction and response assessment of lymphoma patients treated with immunotherapy: a meta-analysis and systematic review
Source: Eur J Nucl Med Mol Imaging. 2022 Aug 6;49(13):4661–76. doi: 10.1007/s00259-022-05918-2 (PMC9606078; doi:10.1007/s00259-022-05918-2)
Supplement: Supplementary file 5 — Supplementary file5 (DOCX 296 KB) [file 259_2022_5918_MOESM5_ESM.docx]

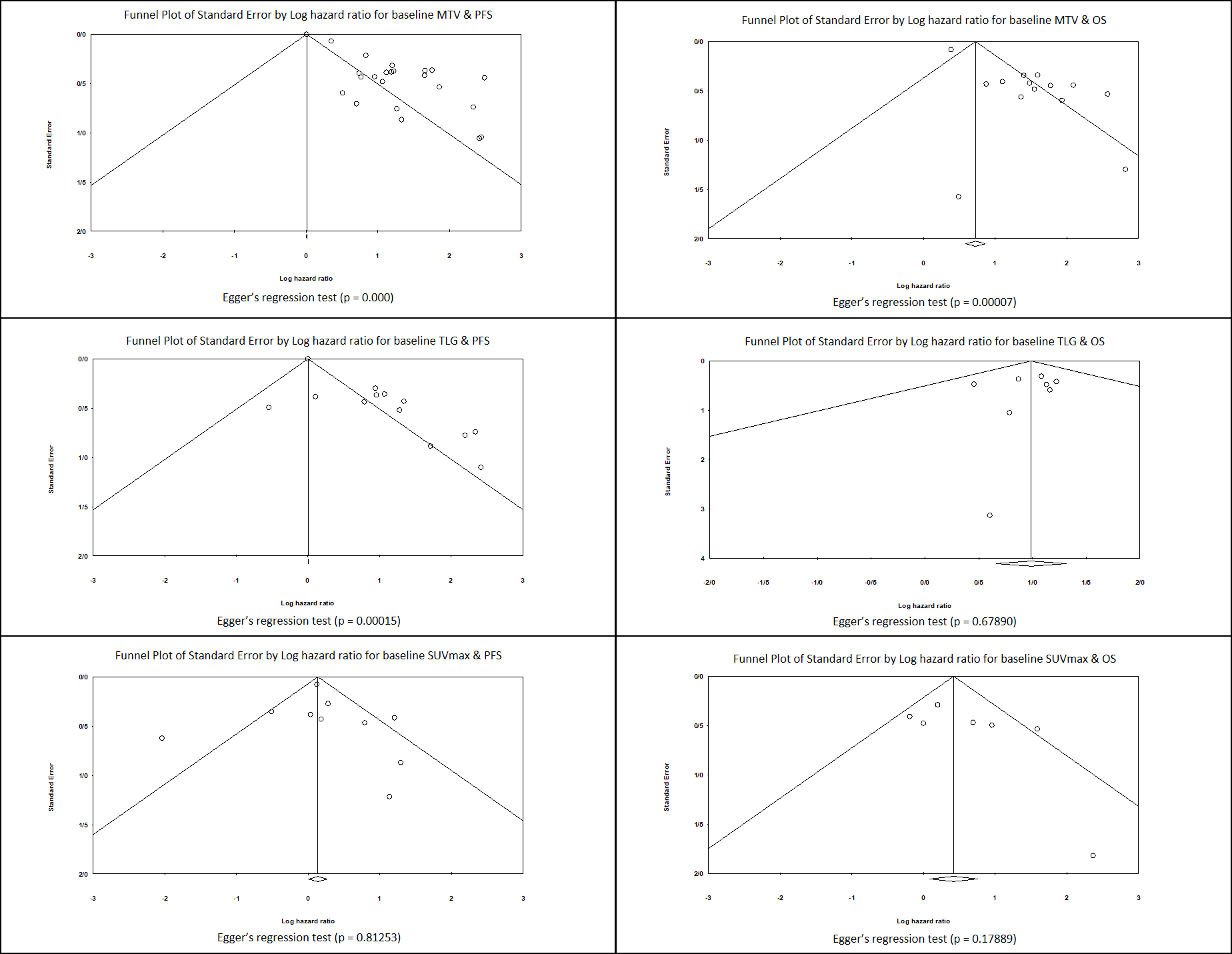


**Figure 1. Funnel plots for publication bias evaluation of reported baseline parameters of enrolled studies on anti-CD20 monoclonal antibodies.**


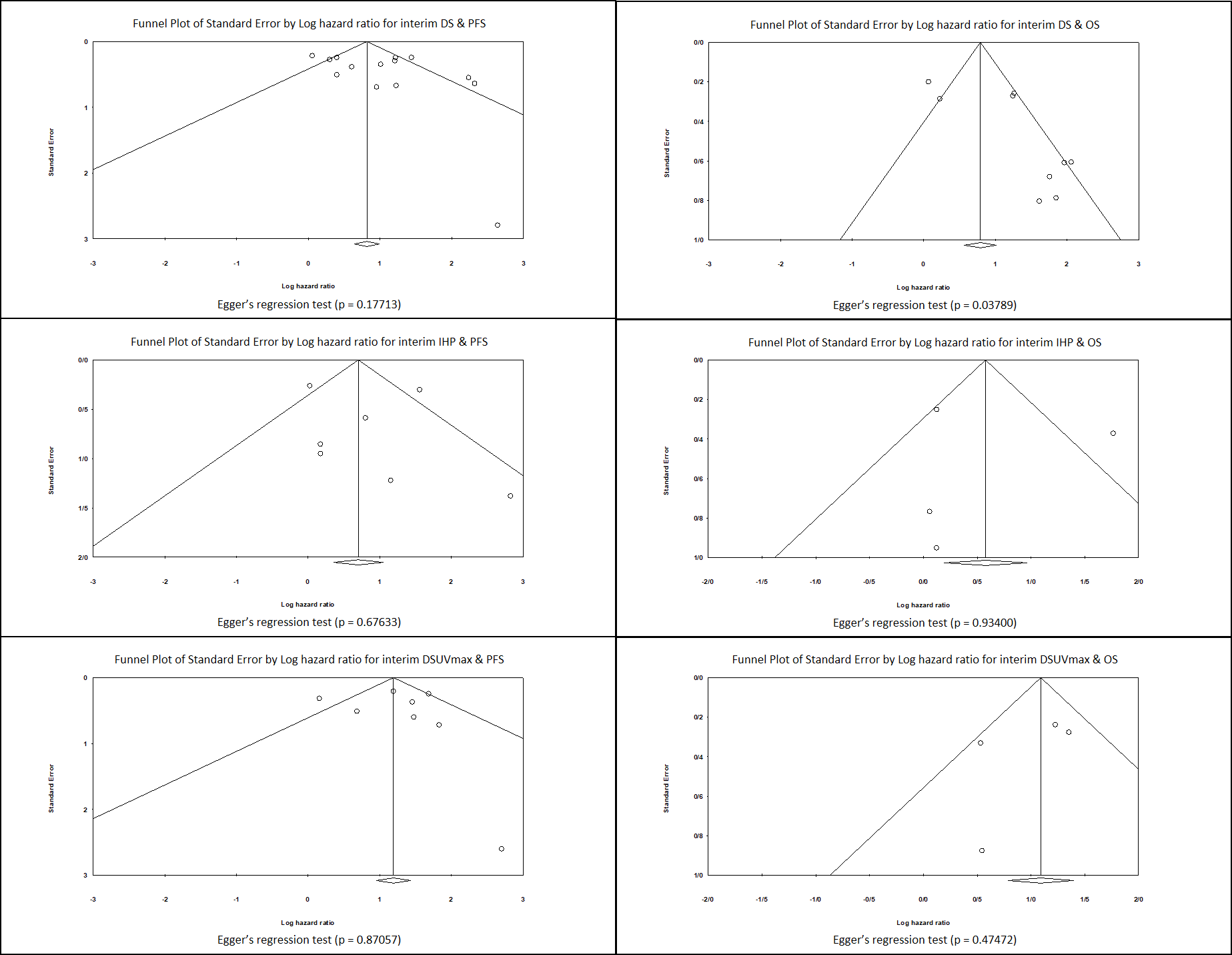


**Figure 2. Funnel plots for publication bias evaluation of reported interim PET parameters of enrolled studies on anti-CD20 monoclonal antibodies.**


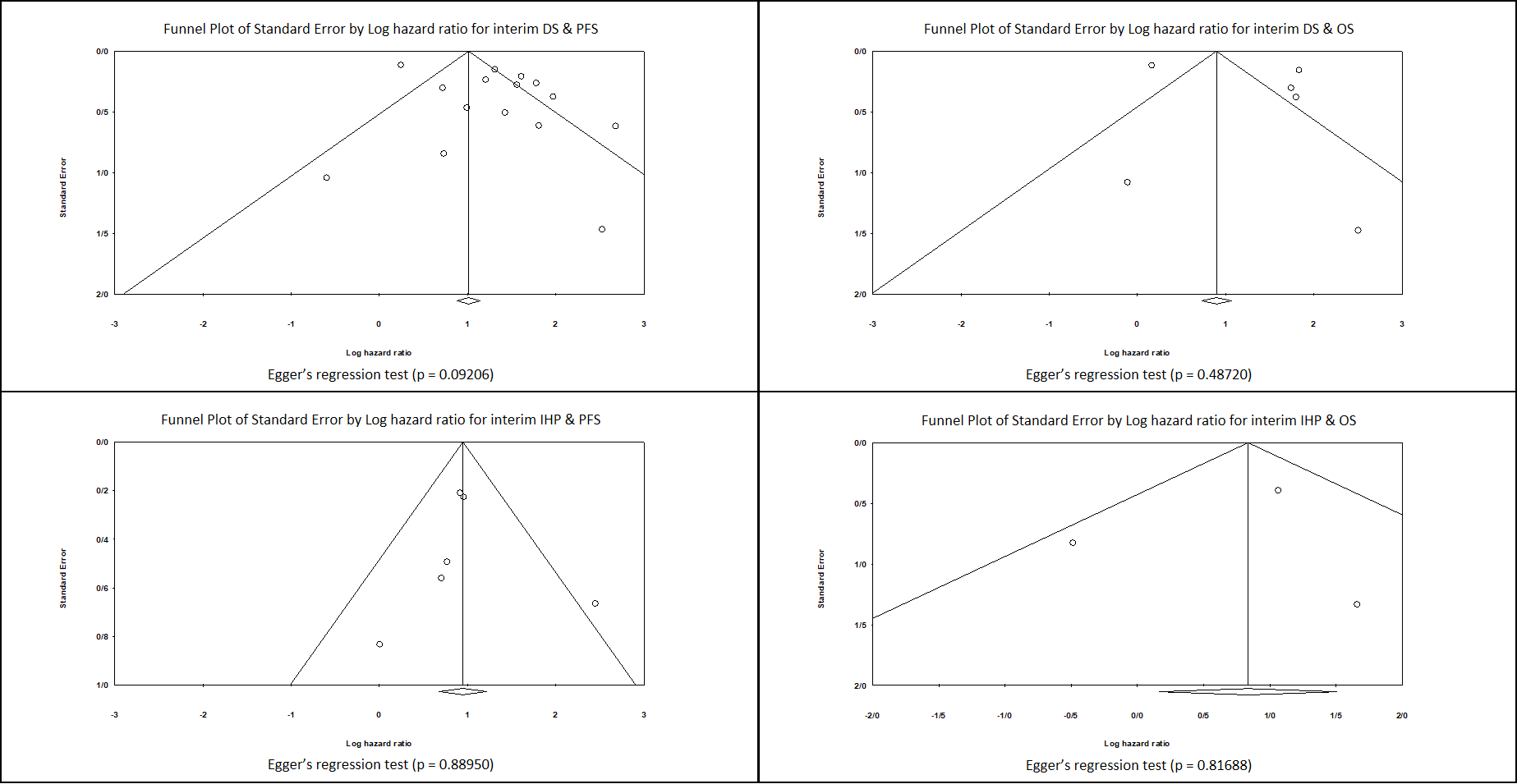


**Figure 3. Funnel plots for publication bias evaluation of reported end of treatment PET parameters of enrolled studies on anti-CD20 monoclonal antibodies.**
